# Duplication of spiralian-specific TALE genes and evolution of the blastomere specification mechanism in the bivalve lineage

**DOI:** 10.1186/s13227-021-00181-2

**Published:** 2021-10-18

**Authors:** Supanat Phuangphong, Jumpei Tsunoda, Hiroshi Wada, Yoshiaki Morino

**Affiliations:** 1 Graduate School of Life and Environmental Sciences, University of Tsukuba, Tsukuba, Ibaraki 305-8572 Japan; 2 College of Biological Sciences, School of Life and Environmental Sciences, University of Tsukuba, Tsukuba, Ibaraki 305-8572 Japan; 3 Faculty of Life and Environmental Sciences, University of Tsukuba, Tsukuba, Ibaraki 305-8572 Japan

**Keywords:** Spiralian development, Gene duplication, Bivalve, Mollusca, TALE homeobox

## Abstract

**Background:**

Despite the conserved pattern of the cell-fate map among spiralians, bivalves display several modified characteristics during their early development, including early specification of the D blastomere by the cytoplasmic content, as well as the distinctive fate of the 2d blastomere. However, it is unclear what changes in gene regulatory mechanisms led to such changes in cell specification patterns. Spiralian-TALE (SPILE) genes are a group of spiralian-specific transcription factors that play a role in specifying blastomere cell fates during early development in limpets. We hypothesised that the expansion of SPILE gene repertoires influenced the evolution of the specification pattern of blastomere cell fates.

**Results:**

We performed a transcriptome analysis of early development in the purplish bifurcate mussel and identified 13 SPILE genes. Phylogenetic analysis of the SPILE gene in molluscs suggested that duplications of SPILE genes occurred in the bivalve lineage. We examined the expression patterns of the SPILE gene in mussels and found that some SPILE genes were expressed in quartet-specific patterns, as observed in limpets. Furthermore, we found that several SPILE genes that had undergone gene duplication were specifically expressed in the D quadrant, C and D quadrants or the 2d blastomere. These expression patterns were distinct from the expression patterns of SPILE in their limpet counterparts.

**Conclusions:**

These results suggest that, in addition to their ancestral role in quartet specification, certain SPILE genes in mussels contribute to the specification of the C and D quadrants. We suggest that the expansion of SPILE genes in the bivalve lineage contributed to the evolution of a unique cell fate specification pattern in bivalves.

**Supplementary Information:**

The online version contains supplementary material available at 10.1186/s13227-021-00181-2.

## Background

Evolutionary developmental studies have described many examples of developmental evolution by altering the expression patterns of conserved toolkit genes [1, 2]. Furthermore, the recent accumulation of genomic information has revealed the presence of many lineage-specific expansions of transcription factors [3]. Although there are several examples in which the developmental role of lineage-specific transcription factors is well characterised, such as bicoid for A-P patterning in flies [4] and duplicated GATA for endoderm and mesoderm specification in some nematodes [5–7], the contribution of lineage-specific transcription factors in the evolution of lineage-specific developmental patterns remains unclear.

Among the three major groups of Bilateria (i.e. Spiralia, Ecdysozoa and Deuterostomia), Spiralia is the largest clade by phyla number, containing almost half of all known metazoan phyla, including large well-known phyla such as Mollusca, Annelida and Platyhelminthes [8]. This animal group is considered sister to Ecdysozoa, which is the other evolutionary branch of Protostomia [9–11]. Despite the stunning diversity of their adult forms, many spiralians display several highly conserved characteristics, known as spiralian development, during their early stages [8, 12, 13]. In addition to the spiral cleavage pattern, individual blastomeres have a similar developmental fate in spiralian development in multiple phyla. The first two cleavages occur vertically along the animal–vegetal axis (the A–V axis) and generate four blastomeres, known as the A, B, C and D quadrants. These usually form the left, ventral, right and dorsal parts of the larva, respectively. Subsequently, horizontal divisions of the blastomere occur at an oblique angle to the A–V axis and create a group of four micromeres, known as a quartet, which are arranged diagonally on top of the lower tier of the macromeres. In general, ectodermal structures develop from the first three quartets, while endodermal organs develop from the fourth quartet and the macromeres [8, 12, 13]. Mesodermal tissues most often arise from two distinct sources: the anterior mesoderm (ectomesoderm) derived from particular cells in the second and third quartets, and the posterior mesoderm (endomesoderm) derived from the mesentoblast 4d cell [8, 12–14]. Another conserved feature is that one of the descendants of the D quadrant (3D, 4d or 2d) possesses organiser activity for the dorsal–ventral axis [8, 12, 13, 15–17].

**Fig. 1:**
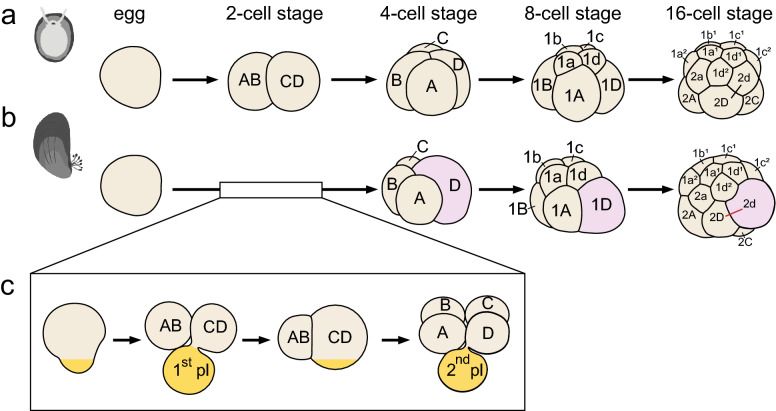
**a, b** Diagram displaying the typical early development of equal-cleaving species (**a**, e.g., limpet) and bivalves (**b**) from the egg stage to the 16-cell stage. **c** Diagram displaying typical polar lobe formation during early bivalve development*.* *pl* polar lobe

Although the developmental pattern is conserved as described above, two major modes of D-quadrant specification can be observed in spiralian development [8, 13, 16, 18]. In many species, the first two cleavages are equal, which causes A–D quadrants to be the same size at the four-cell stage (Fig. 1a). In equal-cleaving species, the D-quadrant fate is specified by inductive interaction usually after the birth of the third quartet [8, 12]. This equal-cleaving embryo with the inductively specified D quadrant has been viewed as an ancestral state of spiralians [19]. By contrast, several spiralian lineages make a larger D cell at the four-cell stage by means of asymmetric cell division or absorption of an extra amount of cytoplasmic content known as the polar lobe (Fig. 1b, c). In these spiralians, the D-quadrant specification is presumed to occur earlier than in equal-cleaving species by cell fate determinants in cytoplasmic contents segregated to D blastomeres [8, 12, 13].

Among molluscs, bivalves exhibit several derived features during early development [20]. During early development, bivalves make unequal-sized blastomeres at two- and four-cell stages by forming a polar lobe at the first and second rounds of cleavage. Consequently, the largest blastomeres, which receive content from the polar lobe, in the two- and four-celled embryos are the CD and D cells, respectively (Fig. 1b, c). In this context, specification of the D quadrant in bivalves has been presumed to occur on the basis of intrinsic components [21, 22].

Another notable feature of early bivalve development is the production of a 2d micromere that is larger than the 2D macromere after division of the 1D macromere [21]. This developmental pattern is in contrast to typical spiral cleavage, in which cell division produces small micromeres at the animal pole and large macromeres at the vegetal pole (Fig. 1a). In gastropods, cells other than those of the 2d lineage also contribute to shell field cells [23–25]. In bivalves, only the 2d lineage contributes to the development of bilaterally separated plates and shell ligaments [20, 26]. However, little is known regarding the genetic mechanisms that specify the blastomere fates of D and 2d blastomeres.

In spiralians, expansions of three-amino acid loop extension (TALE) homeobox genes appear to have occurred several times since the emergence of this animal group, and so far, a number of TALE homeobox genes with cryptic orthology, or orphan genes, have been uncovered in several studies [27–30]. A group of TALE orphan transcription factors originated in the spiralian common ancestor is known as spiralian-TALE (SPILE) [29]. SPILE genes exhibit staggered expression patterns along the A–V axis and play a critical role in the early cell-fate specification of each quartet in the equal-cleaving limpet *Nipponacmea fuscoviridis* [29]. Importantly, numbers of SPILE genes were found to vary among groups of spiralians, suggesting that after the birth of this gene family in the spiralian stem lineage, gene gain and loss events occurred in many lineages [29]. Gene duplication creates redundant genetic materials that can escape from selective pressure, thus offering an opportunity for duplicated genes to become diversified and acquire new functions [31]. Therefore, the duplicated SPILE genes may have acquired new roles in the early specification of blastomere fate in each lineage.

In this study, we examined the relationship between the evolution of the SPILE gene repertoire and modifications in cell-fate establishment in bivalve lineages. We performed a transcriptome analysis of the purplish bifurcate mussel *Mytilisepta virgata* (previously known as *Septifer virgatus*) and identified 13 SPILE genes. We reconstructed the phylogenetic tree of SPILE genes in molluscs and found duplications and diversification of gene sequences in the bivalve lineage. Among those SPILE genes, several genes exhibited lineage-specific expression in the C and D lineages, as well as 2d cell-specific expression in the mussel, implying possible roles for SPILE genes in the cell-fate specification of those cells in bivalves. Overall, our findings suggest that the expansion of bivalve SPILE genes contributed to the evolution of modified cell fate specification patterns in bivalves.

## Results

### Transcriptome analysis and phylogenetic analysis

To reveal the repertoire of the SPILE genes that are expressed in the early developmental stages of bivalves, we conducted transcriptome analysis of multiple early developmental stages (mixture of cleavage, gastrula and trochophore stages) of the mussel *M. virgata*. In the de novo transcriptome assembly, we identified 24 TALE class genes using a BLAST-based search (Additional file 2: Table S1 and S2). To extract SPILE genes, we performed phylogenetic analysis with TALE genes from bilaterian animal lineages and found that 13 genes were classified as SPILE genes (Additional file 1: Fig. S1).

**Fig. 2:**
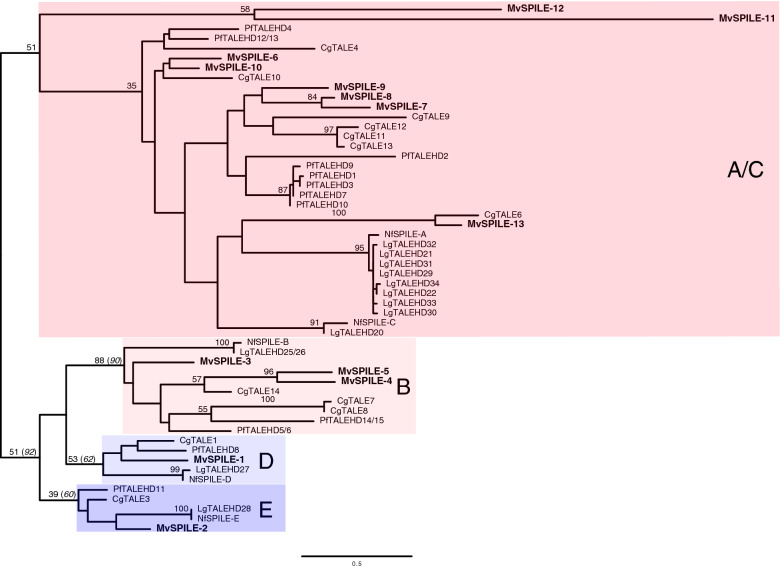
Molecular phylogenetic tree of mollusc SPILE genes. The numbers at the nodes are the bootstrap values (only those ≥ 50% or selected values are shown) obtained using a dataset of all genes and a dataset excluding two rapidly evolving MvSPILE genes (italic, see Additional file 1: Fig. S2). Tree was constructed based on the amino acid sequences of the homeodomains using the maximum likelihood (ML) method. The amino acid substitution model was LG + G + F. Cg: *Crassostrea gigas*, Pf: *Pinctada fucata*, Lg: *Lottia gigantea*, Nf: *Nipponacmea fuscoviridis,* Mv: *Mytilisepta virgata*

To elucidate the evolutionary history of SPILE genes, we constructed a phylogenetic tree using annotated SPILE gene sequences from two bivalves *Pinctada fucata* and *Crassostrea gigas*, and two equal-cleaving gastropod species, the limpet *N. fuscoviridis* and *Lottia gigantea*. (Fig. 2). Based on their phylogenetic relationships, we divided SPILE genes into four clades (B-, D-, E- and A/C-clades; the name of each clade was given based on the NfSPILE genes in that clade). Although the support value for each clade was not high, these values were improved by the removal of two fast-evolving MvSPILE genes—Mv*SPILE-11* and Mv*SPILE-12* (Additional file 1: Fig. S2; Fig. 2). In addition, genes belonging to the same clade shared sequence features outside homeodomains. Each B-clade SPILE gene, as reported previously [29, 30], contains two homeodomains (Additional file 1: Fig. S3). Although a previous study has classified the D-clade SPILE genes of bivalves and limpets into separate clades, ([30], Additional file 2: Table S2), we found that the sequences outside the homeodomain were also similar among the D-clade genes, (Additional file 1: Fig. S4), supporting the close relationships between D-clade SPILE genes. SPILE genes in the A/C-clade have been classified into various gene clades in a previous study ([30], Additional file 2: Table S2). On the other hand, most of these genes reportedly have a PADRE domain, a conserved region of approximately 90 amino acids that lies upstream of the homeodomain ([27, 30] Additional file 2: Table S2). Actually, most of the A/C-clade SPILE genes, including mussel genes, have a region similar to the PADRE domain upstream of the homeodomain (Additional file 1: Fig. S5; Additional file 2: Table S2). Concerning TALE genes in molluscs, the existence of a PADRE domain outside the A/C-clade was reported only for Nf*SPILE-D* ([30]; Additional file 2: Table S2). However, we found that all other D-clade genes and most of the B-clade genes have a region similar to the PADRE domain upstream of the homeodomain, although the corresponding regions are slightly longer than the PADRE domain in the A/C-clade genes (Additional file 1: Fig. S6). We did not find conserved regions that corresponded to the PADRE domain in the E-clade SPILE genes (Additional file 1: Fig. S7).

Based on this phylogenetic framework, the results showed that single SPILE-D and E clade genes were present in the examined species (Table 1). Conversely, in the B-clade, one SPILE gene was found in each limpet, whereas two to three genes were identified in the bivalves, suggesting that duplication events had occurred in the bivalve lineage. The number of SPILE A/C-type genes found in the limpet *Lottia gigantea* and bivalve species was similar (Table 1). However, eight homeodomain sequences in the limpet (Lg*TALEHD 21*, *22*, and *29*–*34*) were nearly identical and located tandemly in the genome ([29]; Fig. 2; Additional file 2: Table S2), suggesting recent and lineage-specific duplications of these homeobox sequences. Conversely, seven or eight SPILE genes in the A/C clade have been predicted in each bivalve species, and many of these genes are not in tandem in the genome ([29]; Additional file 2: Table S2).

**Table 1:** The number of SPILE genes of each clade in limpet and bivalve

Clade	Sequence features	Limpet (equal)		Bivalve (Unequal)		
		*N. fuscoviridis* (transcriptome)	*L. gigantea* *(*genome)	*C. gigas* (genome)	*P. fucata* (genome)	*M. virgata* (transcriptome)
A/C	PADRE domain	2	9	7	8	8
B	two homeodomains modified PADRE domain	1	1	3	2	3
D	Highly conserved sequence outside homeodomain modified PADRE domain	1	1	1	1	1
E	No PADRE domain	1	1	1	1	1

The number of genes in *L. gigantea* included homeodomain sequences that were not predicted to be genes

**Fig. 3:**
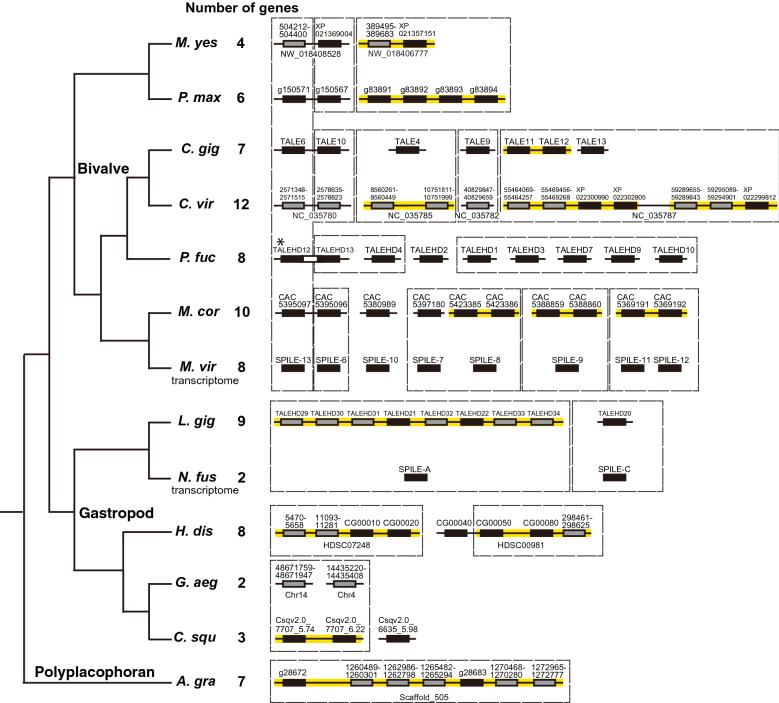
The number and genomic locations of A/C-clade SPILE genes in molluscs. The number of genes included homeodomain sequences that were not predicted to be genes. The phylogenetic relationships were based on previous studies [34, 58, 59]. Black squares on the line: predicted gene models. Grey squares on the line: SPILE-A/C clade homeodomains extracted from the genomic sequence (not predicted to be genes). Black squares: genes predicted by transcriptomic analysis. Genes located in the same scaffold/chromosome sequence that form a clade in phylogenetic analysis (bootstrap values ≥ 80% in Additional file 1: Fig. S9–12) are highlighted in yellow. Genes in a clade supported by bootstrap values ≥ 50% (Additional file 1: Fig. S9–12) are indicated by dashed lines. * Gene model pfu_aug2.0_460.1_00771.t1 included two distinct homeodomains (PfTALEHD12 and 13; Additional file 2: Table S2). *M*. *yes*: *Mizuhopecten yessoensis*, *P. max*: *Pecten maximus*, *C*. *gig*: *Crassostrea gigas*, *C. vir*: *Crassostrea virginica*, *P. fuc*: *Pinctada fucata*, *M. cor*: *Mytilus coruscus, M. vir*: *Mytilisepta virgata*, *L. gig*: *Lottia gigantea*, *N. fus*: *Nipponacmea fuscoviridis, H. dis*: *Haliotis discus hannai*, *G. aeg*: *Gigantopelta aegis*, *C. squ*: *Chrysomallon squamiferum*, *A. gra*: *Acanthopleura granulata*

To gain further insight into the timing of duplication of the A/C-clade SPILE genes, we extracted SPILE sequences from additional molluscan species, including one chiton species (*Acanthopleura granulata* [32]), three gastropod species (abalone *Haliotis discus hannai* [33]*,* deep-sea hydrothermal vent snails *Gigantopelta aegis* [34] and *Chrysomallon squamiferum* [35]), and the four bivalve species (hard-shelled mussel *Mytilus coruscus* [36], the eastern oyster *Crassostrea virginica* [37], the Japanese scallop *Mizuhopecten yessoensis* [38], and the king scallop *Pecten maximus* [39]. Among the three gastropods, abalone is an equal-cleaving mollusc [40]; however, no information on the embryonic development of the other two species is available. Although no information is available on the cleavage pattern of the chiton species examined (*A. granulata*), several chiton species show equal cleavage in the first two divisions [41–43]. The A/C-clade homeobox sequences from these species were extracted by combining BLAST and phylogenetic analysis (Additional file 1: Fig. S8–10; Additional file 2: Table S2; see the “Methods” section for details). Furthermore, to increase the resolution of phylogenetic relationships in the A/C-clade SPILE genes, phylogenetic analysis without B/E-clade SPILE genes (D-clade SPILE genes were used as an outgroup) was performed (Additional file 1: Fig. S11, using sequences of molluscs; Additional file 1: Fig. S12, using sequences of bivalves). Although many clades composed of SPILE genes from the same or closely related species were recovered with bootstrap values ≥ 50, most clades containing SPILE genes from distantly related species were not recovered with bootstrap values ≥ 50 (Additional file 1: Fig. S9–12). Only the clade containing MvSPILE-13 and CgTALE-6 (TALE-V clade; [30]) was supported with high bootstrap values as a clade composed of SPILE genes from distant bivalve species (Additional file 1: Fig. S9–12). The number of A/C-clade genes (including homeodomain sequences which are not predicted as genes), position on the genome, and clades with bootstrap values ≥ 50 are summarised in Fig. 3.

Although the SPILE-A/C gene duplications were also found in chitons and gastropods, most duplications likely occurred in each lineage independently (highlighted in yellow in Fig. 3). For example, although seven SPILE-A/C clade genes were identified in chiton, all of them were located on the same scaffold and had nearly identical homeobox sequences, suggesting that one type of homeobox gene was duplicated in the chiton lineage (Fig. 3). In abalone, eight A/C-clade genes were identified, and four genes were closely located on the two scaffolds (Additional file 2: Table S2; Fig. 3, HDSC07248 and HDSC00981). Two and three SPILE-A/C genes were identified in the two deep-sea gastropods, respectively, and the two genes in C. *squamiferum* located in the same scaffold and homeodomain sequence were identical, indicating a recent duplication event had occurred. These results suggest that most of the duplications observed in the A/C-clade SPILE genes in gastropods and chiton occurred independently in each lineage and that the ancestors of molluscs may have 1–2 types of SPILE genes in the A/C-clade.

In bivalves, although the number of A/C-clade SPILE genes varied widely from 4 to 12 and there were potential lineage-specific duplications (Fig. 3; highlighted in yellow), diverse types of homeodomains were found in all species examined. For example, in the scallop (*M. yessoensis*) that had the smallest number (four) of A/C-clade SPILE genes among the examined bivalve species, three types of distinct homeobox sequences were found (Additional file 1: Fig. S9–12; Fig. 3; e.g. My_NW_018408528.1_504212-504400, My_XP_021369004.1, and My_XP_021357151.1/My_NW_018406777.1_389495-389683). Similarly, more than three types of homeobox sequences were identified in the other bivalves examined (Additional file 1: Fig. S9–12; Fig. 3). These results suggest that duplications and subsequent sequence divergence of the SPILE A/C-clade genes in the bivalve lineages had occurred.

### Expression patterns of MvSPILE genes

To explore the roles of SPILE genes in the early development of bivalves, we examined the expression patterns of 12 SPILE genes in the mussel, with the exception of Mv*SPILE-13*, for which only a short fragment of the gene was recovered (Additional file 3: Dataset S1).

### MvSPILE genes in D- and E- clades

**Fig. 4:**
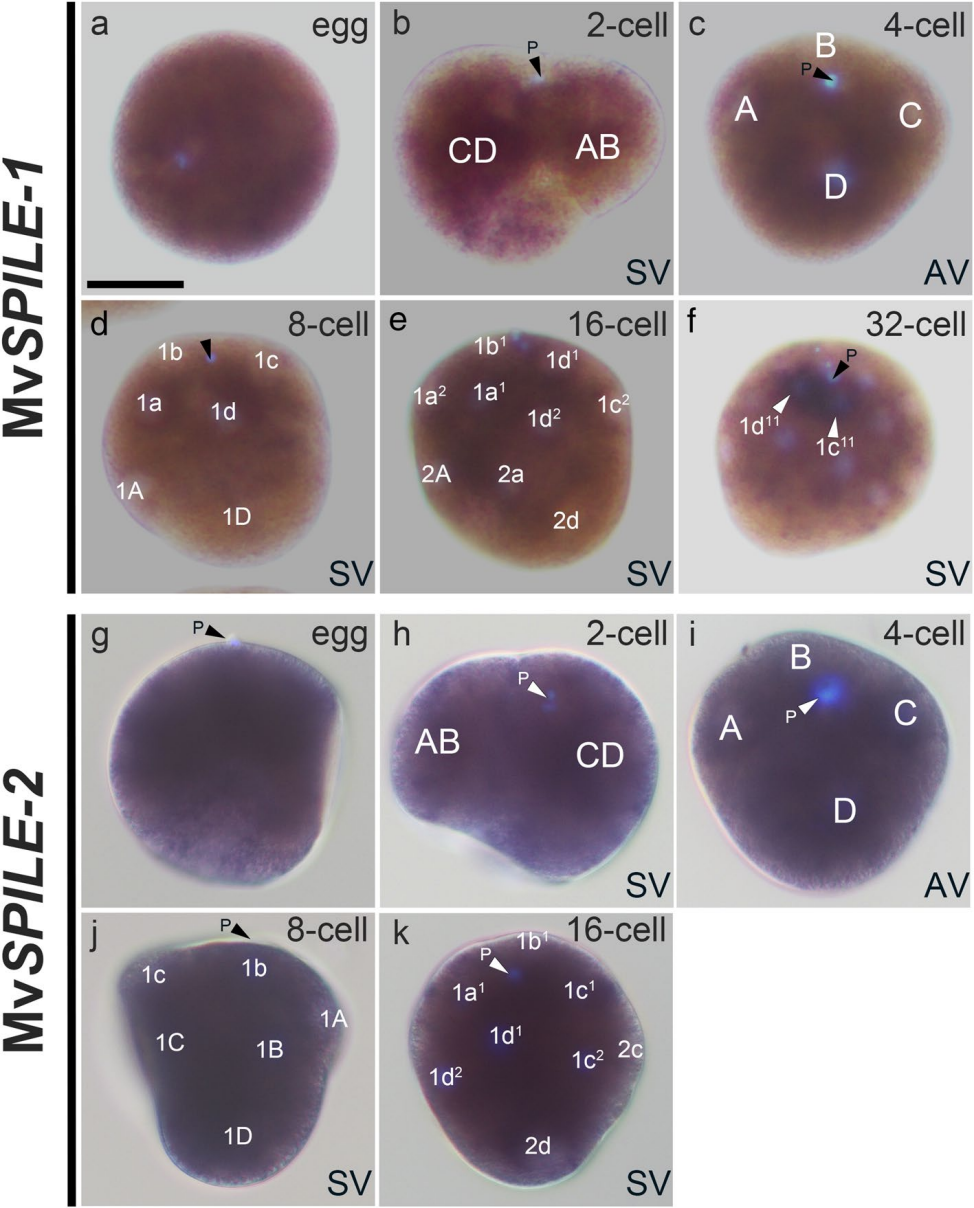
Expression patterns of MvSPILE genes in the D- and E- clade, including Mv*SPILE-1* (**a**–**f**) and Mv*SPILE-2* (**g–k**). DNA was stained using DAPI (light blue). Polar bodies are indicated by arrowheads with the letter ‘P’. *SV* side view, *AV* animal view. Scale bar indicates 50 μm

Mv*SPILE-1* and Mv*SPILE-2*, in the D and E clades are expressed maternally (Fig. 4a, g). The counterparts of these two genes in limpets, Nf*SPILE-D* and Nf*SPILE-E*, were also reported to exhibit maternal expression patterns [29]. In limpets, the Nf*SPILE-D* gene is expressed in all blastomeres until the eight-cell stage, but expression is restricted to the four blastomeres at the animal pole at the 16- and 32-cell stages. Similarly, Mv*SPILE-1* was expressed uniformly in all blastomeres until the 16-cell stage, but was expressed predominantly in the two animal-most blastomeres, 1c^11^ and 1d^11^, at the 32-cell stage (Fig. 4b–f; Additional file 1: Fig. S13) By contrast, unlike limpet Nf*SPILE-D*, expression was predominantly restricted to the C and D blastomeres among 1q^11^ lineages (Fig. 4f; Additional file 1: Fig. S13). Mv*SPILE-2* was expressed in all blastomeres until the 16-cell stage (Fig. 4g–k; Additional file 1: Fig. S13), similar to its limpet counterpart gene [29].

### MvSPILE genes in B-clade

**Fig. 5:**
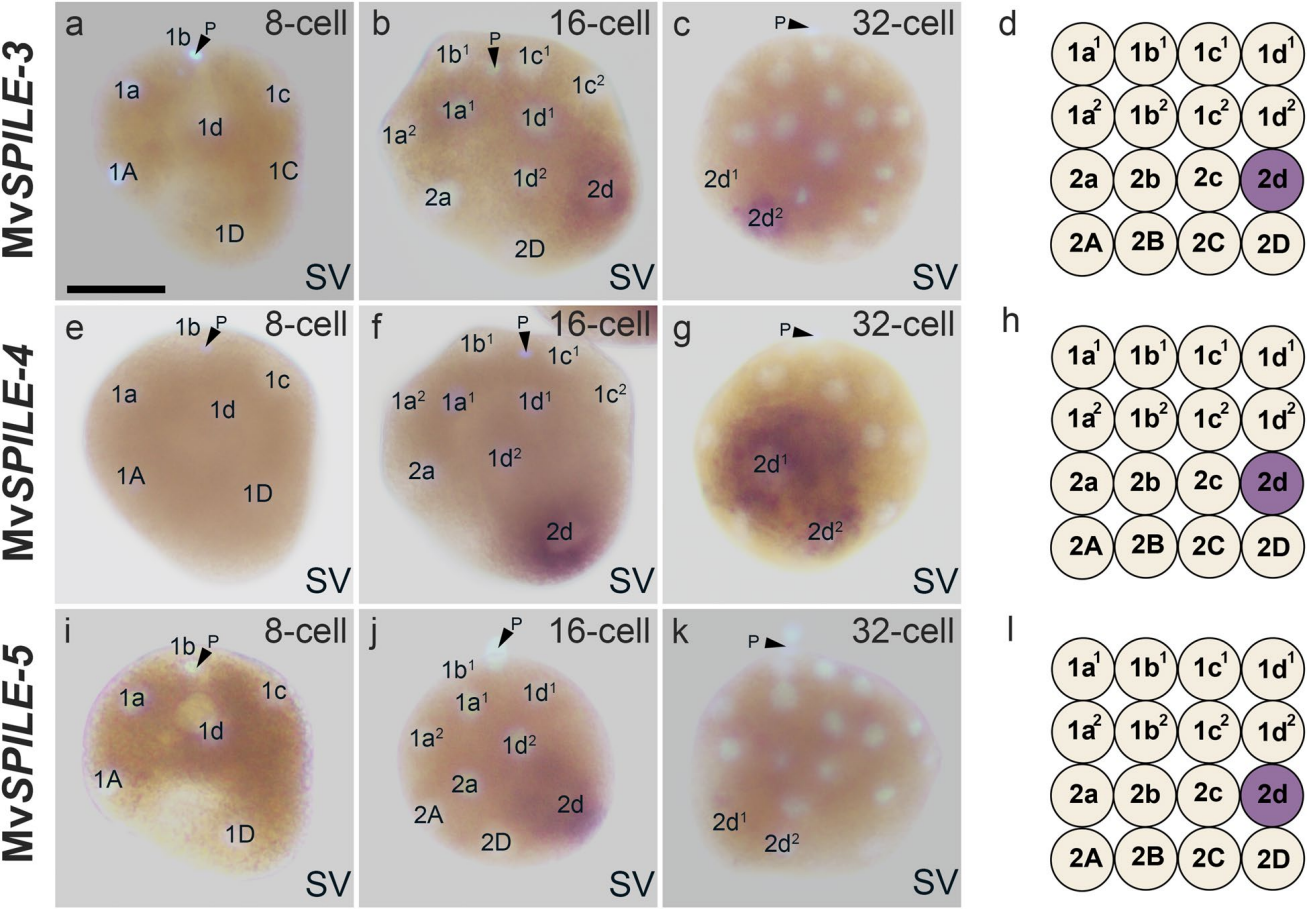
Expression patterns of MvSPILE genes in the B-clade, including Mv*SPILE-3* (**a**–**d**), Mv*SPILE-4* (**e**–**h**) and Mv*SPILE-5* (**i**–**l**). DNA was stained using DAPI (light blue). Polar bodies are indicated by arrowheads with the letter ‘P’. The expression patterns of each gene are illustrated in diagrams on the right (**d**, **h**, and **l**, 16-cell). SV: side view. Scale bar indicates 50 μm

Although only one gene belonging to the SPILE-B clade was found in limpets (Fig. 2; [29]), three SPILE-B genes were found in the mussel transcriptome. All three genes, MvS*PILE-3*, *-4*, and *-5*, showed expression in 2d blastomeres at the 16-cell stage (Fig. 5a, b, d, e, f, h, i, j, l). In limpets, Nf*SPILE-B* expression was detected in the second quartet at the 16-cell stage, but its expression was not restricted to the D lineage [29], which differed from the findings in bivalves. All B-clade SPILE genes were initially expressed in the 2d blastomere at the 16-cell stage, but their expression patterns diverged later at the 32-cell stage (Fig. 5c, g, k). Mv*SPILE-3*, the signal was restricted to the 2d^2^ blastomere (Fig. 5c), whereas the signal of Mv*SPILE-4* was present in both 2d daughter cells (2d^1^ and 2d^2^; Fig. 5g), and no specific expression of Mv*SPILE-5* was detected at the 32-cell stage (Fig. 5k). These expression patterns imply that functional differentiation has occurred between Mv*SPILE-3*, -*4*, and *-5*.

### MvSPILE genes in A/C-clade

**Fig. 6:**
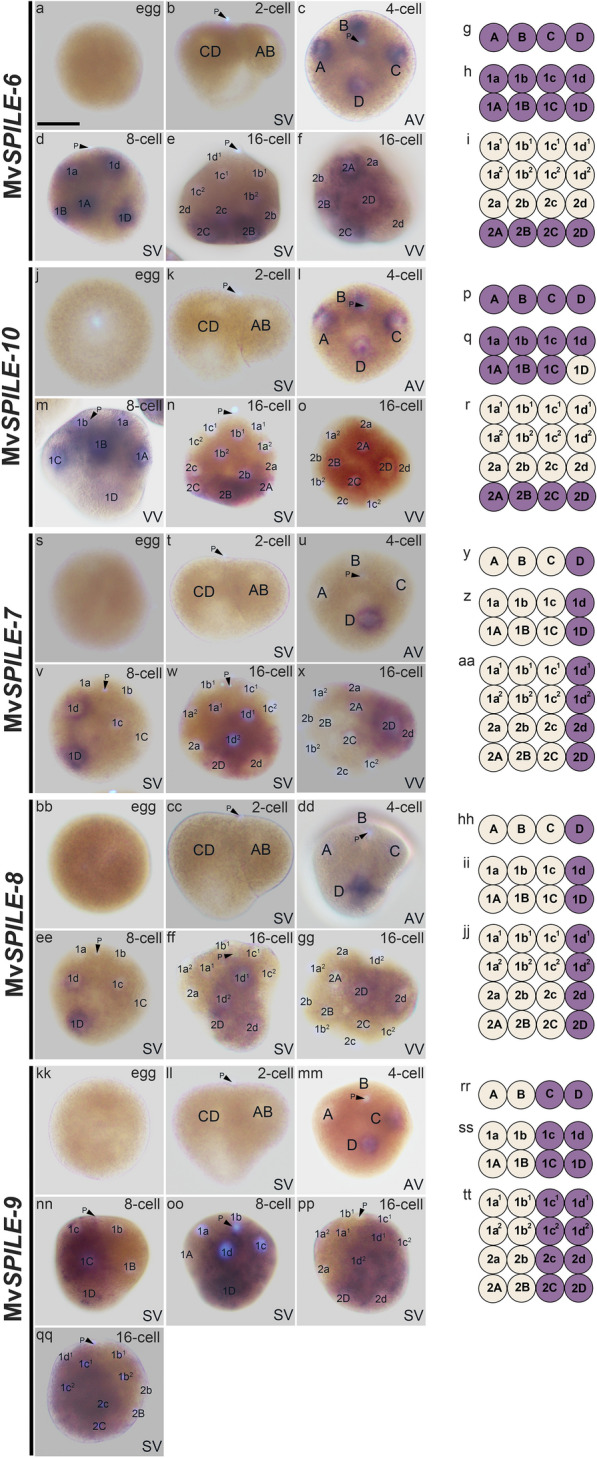
Expression patterns of MvSPILE genes in the A/C-clade, including Mv*SPILE-6* (**a**–**i**), Mv*SPILE-10* (**j**–**r**), Mv*SPILE-7* (**s**–**aa**), Mv*SPILE-8* (**bb**–**jj**), and Mv*SPILE-9* (**kk**–**tt**). DNA was stained using DAPI (light blue). Polar bodies are indicated by arrowheads with the letter ‘P’. **n** and **o**, **w** and **x**, **ff** and **gg**, **nn** and **oo**, **pp** and **qq** are different individuals of the same stage, respectively. The expression patterns of each gene are illustrated in diagrams on the right (**g**, **p**, **y**, **hh**, and **rr**, 4-cell; **h**, **q**, **z**, **ii**, and **ss**, 8-cell; **i**, **r**, **aa**, **jj**, and **tt**, 16-cell). *SV* side view, *AV* animal view, *VV* vegetal view. Scale bar indicates 50 μm

Transcriptome analysis of the early developmental stage identified two SPILE genes (Nf*SPILE-A* and *-C*) in limpets [29], whereas eight SPILE genes were identified in mussels. One of those, Mv*SPILE-6* was expressed in all blastomeres at the 4- and 8-cell stage, but exhibited macromere-specific expression at the 16-cell stages (Fig. 6a–i). Similarly, Mv*SPILE-10* expression was first detected in all blastomeres at 4-cell stage (Fig. 6j-k, p). However, at the 8-cell stage, signals of the Mv*SPILE-10* gene appeared to be specifically excluded from the 1D blastomere (Fig. 6m, q). Nevertheless, at the 16-cell stage, Mv*SPILE-10* signals were again evident in the 2D cell along with the other macromeres, similar to Mv*SPILE-6* (Fig. 6n, o, r).

In contrast, three of the other A/C-clade MvSPILE genes exhibited quadrant-specific expression patterns at the 4-cell stage. For example, Mv*SPILE-7* and Mv*SPILE-8* were expressed in the D blastomere at the 4-cell stage (Fig. 6s–u and y; bb-dd and hh). In addition, Mv*SPILE-9* appeared to be expressed in the C and D blastomeres at the 4-cell stage (Fig. 6 kk-mm and rr). These SPILE genes were expressed in all daughter cells of D-quadrant (Mv*SPILE-7* and *-8*) or C- and D-quadrants (Mv*SPILE-9*) at the 8- and 16-cell stages (Fig. 6 v-x, z and aa; ee-gg, ii and jj; nn-qq, ss, and tt).

**Fig. 7:**
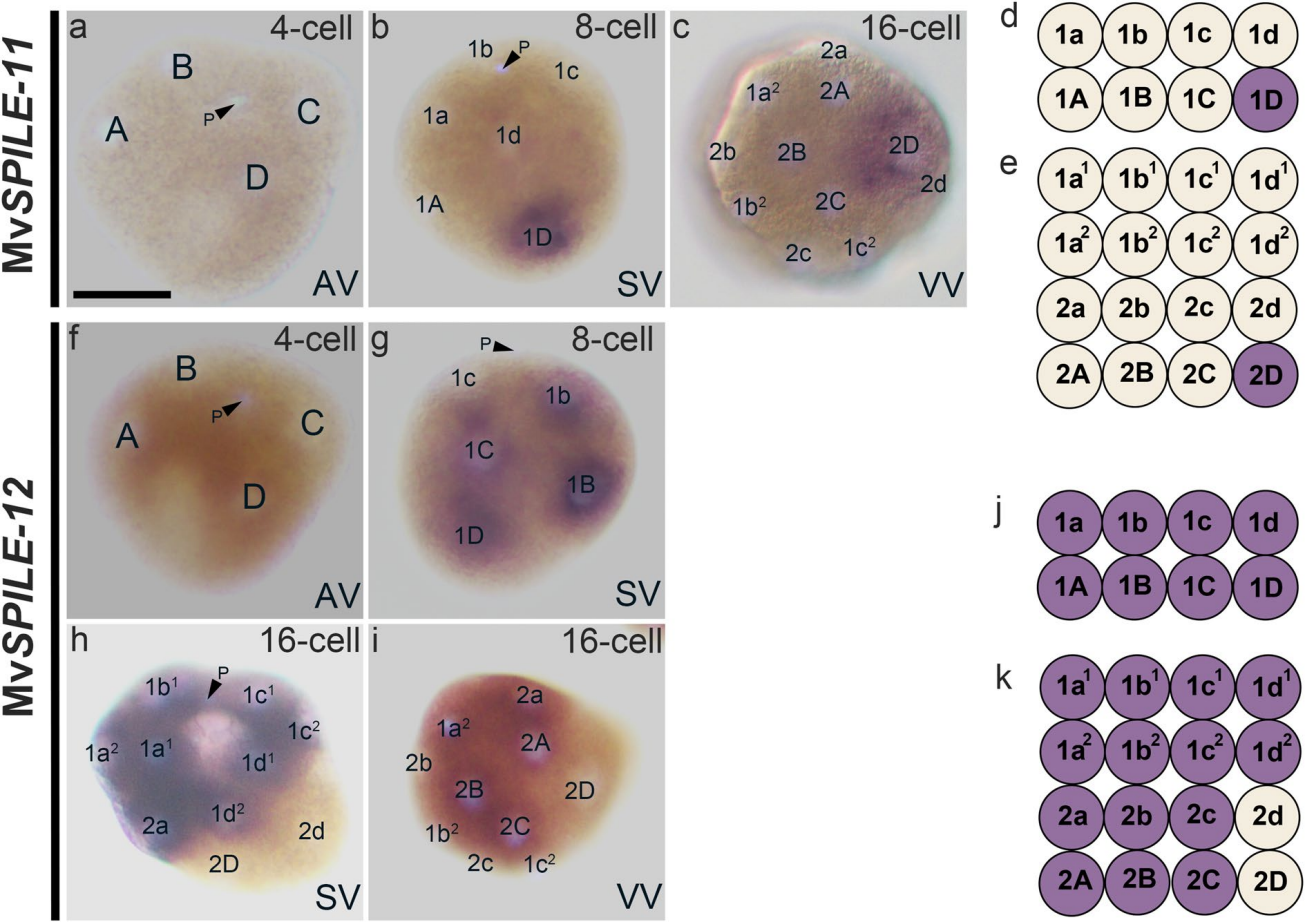
Expression patterns of MvSPILE genes in the A/C-clade, including Mv*SPILE-11* (**a**–**e**) and Mv*SPILE-12* (**f**–**k**). DNA was stained using DAPI (light blue). Polar bodies are indicated by arrowheads with the letter ‘P’. **h** and **i** are different individuals of the same stage. The expression patterns of each gene are illustrated in diagrams on the right (**d** and **j**, 8-cell; **e** and **k**, 16-cell). *SV* side view, *AV* animal view, *VV* vegetal view. Scale bar indicates 50 μm

**Fig. 8:**
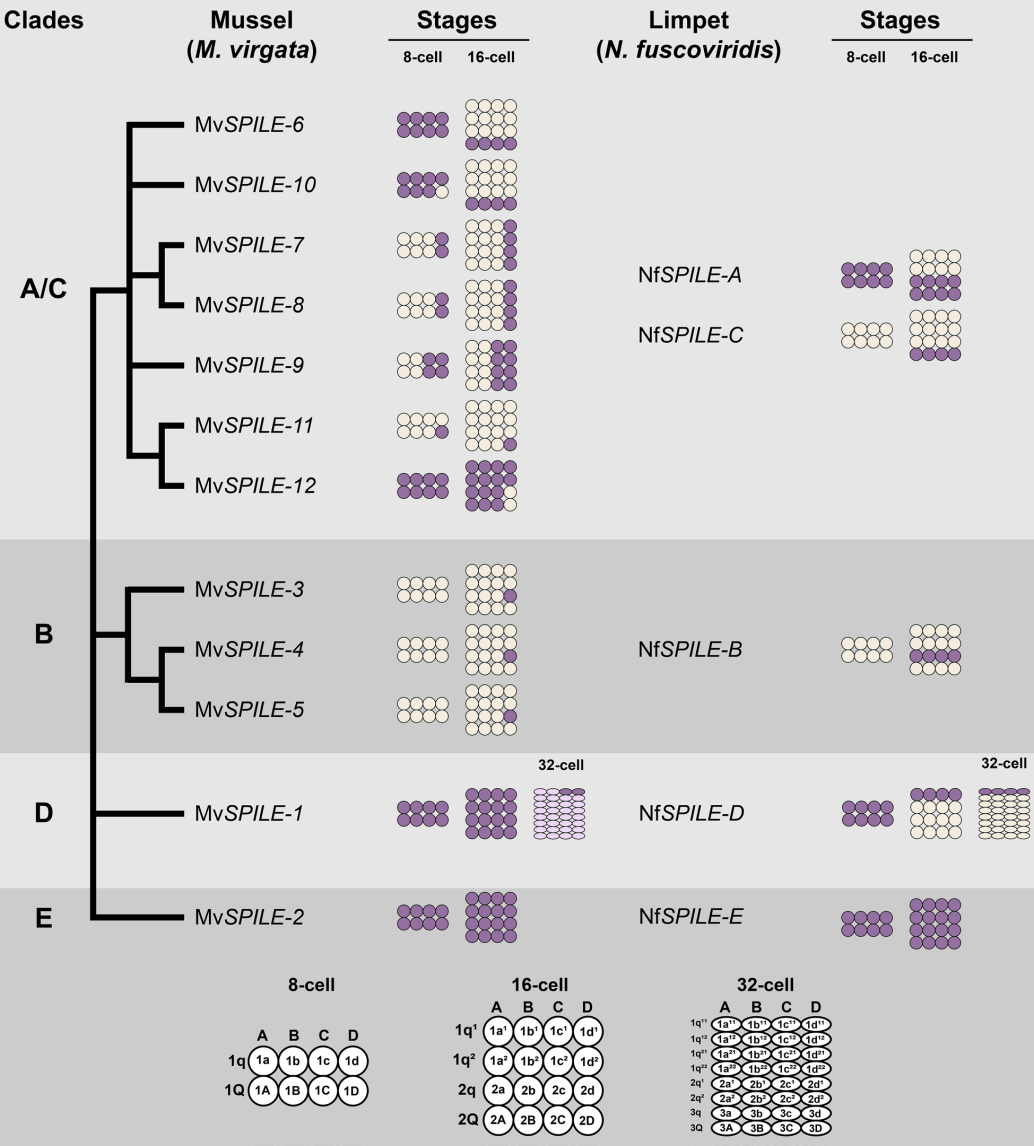
Summary diagram showing phylogenetic relationships as well as the expression patterns of twelve SPILE genes in mussel (*Mytilisepta virgata*) and five SPILE genes in limpet (*Nipponacmea fuscoviridis*)

Two of the remaining genes were expressed/not expressed only in certain blastomeres. The expression of Mv*SPILE-11* was first detected in the 1D blastomere at the eight-cell stage, and the signal was restricted to the 2D cell at the 16-cell stage (Fig. 7a–e). The expression of Mv*SPILE-12* was detected in all blastomeres at the eight-cell stage, but it was no longer expressed in the 2d and 2D cells at the 16-cell stage, while remaining in all other cells (Fig. 7f–k). We summarised the expression patterns and phylogenetic relationships of SPILE genes in limpets and mussels (Fig. 8).

## Discussion

### Some SPILE genes may function in the specification of the C and D quadrants and the 2d blastomere in bivalves

In addition to the conserved features of spiralian development, the early developmental processes of bivalves exhibit derived features. One of these features is that the early specification of the D blastomere begins with the segregation of polar lobes. Another characteristic of bivalve development is the formation of a larger 2d blastomere, which derives the bivalve shell and ligament anlage [20, 26]. To explore the genetic background of those early developmental events, we examined the evolutionary history of SPILE genes in molluscs and the expression pattern of 12 SPILE genes in bivalves.

In the limpet *N. fuscoviridis*, four of five SPILE genes (Nf*SPILE-A*–*D*) exhibited staggered expression patterns along the A–V axis and function in the specification of each quartet lineage (Fig. 8; [29]). In bivalves, two SPILE genes (Mv*SPILE-6* and Mv*SPILE-10*) exhibited macromere-specific expression patterns at the 16-cell stage (Fig. 6e and f; n and o), implying that these SPILE genes contribute to quartet specification.

In contrast, we found that two SPILE genes, Mv*SPILE-7* and Mv*SPILE-8*, showed D-blastomere-specific expression at the 4-cell stage (Fig. 6u and dd), which led us to speculate that the expression of these genes is controlled by the polar lobe content. However, it is necessary to observe the gene expression of these SPILE genes after removal of the polar lobe in order to clarify whether the expression is controlled by the polar lobe. These two genes were also expressed in all daughter cells of the D blastomere at the 8- and 16-cell stages (Fig. 6v–x and ee–gg). Based on these expression patterns, we suggest that these two SPILE genes are components of a gene regulatory network for the specification of the D quadrant and subsequent secondary axis formation.

Furthermore, Mv*SPILE-9* was expressed in both the C and D blastomeres and its descendants at 4–16 cell stages (Fig. 6mm–qq). Therefore, not only D blastomeres but also C blastomeres are distinct from A and B blastomeres from the 4-cell stage, based on gene expression patterns. The early specification of C and D blastomeres is supported by the predominant expression of Mv*SPILE-1* 1c^11^ and 1d^11^ at the 32-cell stage (Fig. 4f; Additional file 1: Fig. S13). In snails, expression of the *nodal* gene, which determines the left–right axis, begins in C-blastomere descendants at the 32-cell stage [44]. The C blastomere may also be important for axis formation in bivalves.

Production of a larger 2d micromere at the 16-cell stage is another derived feature present in bivalve development. This 2d lineage gives rise to several major structures including the posttrochal epidermis of the larva, as well as the bilateral shell field and shell ligament [26]. Among MvSPILE genes, we found that the expression of three genes of the B clade, Mv*SPILE-3*, Mv*SPILE-4* and Mv*SPILE-5*, was confined to the 2d cell and some of its derivatives (Fig. 5). Thus, these MvSPILE genes are maybe responsible for the establishment of 2d cell identity and the segregation of cell fate within the 2d lineage.

In summary, two SPILE genes in bivalves exhibited quartet-specific expression, and some genes exhibited expression specific to the C, D quadrants, as well as 2d blastomere. Although functional analysis is necessary to demonstrate their function, these expression patterns suggest that SPILE genes are involved in the specification of D blastomere at the four-cell stage and in 2d blastomere, both of which are derived features in bivalve development.

### Rapid changes in the expression of SPILE genes in the course of embryonic development of mollusc

Several SPILE genes (e.g., Mv*SPILE-6, -10*, *-11* and *-12*) exhibited changes in expression pattern among stages of development. These rapid changes in expression might have been due to unequal mRNA segregation and/or rapid RNA degradation, in addition to transcriptional regulation. It is unclear which process is more important, but at least some SPILE expression cannot be explained by unequal segregation alone. For example, Mv*SPILE-12* signals were detected in the 1D blastomere at the eight-cell stage (Fig. 7g), but no expression was detected in the 1D descendant blastomeres, 2d and 2D, at the 16-cell stage (Fig. 7h, i). This absence of expression in 1D descendant blastomeres cannot be explained by unequal RNA segregation alone. This degree of rapid shift in the expression of SPILE genes, possibly due to rapid RNA degradation, is also observed in limpets [29]. Although further analysis of the localisation mechanism is necessary, these findings imply that localisation on the basis of mRNA degradation is an ancestral property of SPILE genes in molluscs.

### SPILE gene duplication and expression pattern diversification in bivalves

Based on the phylogenetic analysis in the present study, we found that mollusc SPILE genes were grouped into four major clades, and the duplications of SPILE A/C- and B-clade genes occurred in the bivalve lineage (Table 1; Fig. 3). Although duplications of the SPILE-A/C gens were observed in other molluscan lineages such as gastropod and chiton, phylogenetic analysis and locations of SPILE genes on genome suggest that duplications have occurred in each lineages independently (Fig. 3; Additional file 1: Fig S9–12).

The patterns of SPILE expression were similar in bivalves and limpets in that the SPILE genes in the D- and E-clades were expressed maternally (Fig. 4a, g; [29]). Furthermore, SPILE genes in the A/C- and B-clades were similar in that zygotic expression was initiated at the 4–16-cell stages (Figs. 5, 6, 7; [29]). However, there were differences in the expression of SPILE genes between limpets and bivalves. The expression patterns of Mv*SPILE-1* and Nf*SPILE-D* were similar in that they were predominantly expressed in most animal blastomeres (Fig. 4f; Additional file 1: Fig. S13 [29]). However, the patterns differed in that Nf*SPILE-D* were expressed in all cells of the 1q^11^ lineage at 32-cell stage in limpets, but predominant expression of Mv*SPILE-1* was restricted to the 1c^11^ and 1d^11^ blastomeres in bivalves (Fig. 4f; Additional file 1: Fig. S13; [29]). Moreover, SPILE genes in the A/C- and B-clades exhibited several divergent expression patterns. All B-clade SPILE genes showed 2d-specific expression at the 16-cell stage (Fig. 5). Furthermore, three A/C-clade SPILE genes showed expression of D-quadrant or C- and D-quadrant-specific expression from the 4-cell to 16-cell stage (Fig. 6). These expression patterns were clearly distinct from the expression patterns of their limpet counterparts (Fig. 8; [29]). In the polychaete *Spirobranchus kraussii*, three SPILE genes exhibited staggered expression patterns along the A–V axis [29]. These observations suggest that SPILE genes had staggered expression along the A–V axis in the most recent common ancestor of annelids and molluscs, and that quadrant-specific and blastomere-specific expression patterns were acquired in the bivalve lineage.

We questioned whether the divergence of the expression pattern of the SPILE gene is associated with gene duplication. As for the B-clade, all mussel genes are expressed in 2d blastomere (Fig. 5). Therefore, it is likely that the acquisition of expression in the 2d blastomere may have occurred before gene duplication. For the A/C clade, several genes (Mv*SPILE-7*, *-8*, and *-9*) are expressed in the D or in the C and D quadrants, whereas several genes (Mv*SPILE-6* and *-10*) retained a possible ancestral quartet-specific expression pattern (Fig. 6). Therefore, we propose an evolutionary scenario in which duplications of SPILE A/C clade genes contributed to the evolution of cell-fate specification patterns, such as C- and D-quadrant-specification at the four-cell stage in the bivalve lineage. Our results imply that the expansion of transcription factors may have facilitated the evolution of lineage-specific developmental patterns.

Due to the low resolution of the phylogenetic relationships of the SPILE genes of bivalves and lack of spatial expression data on SPILE genes in bivalves other than mussels, it remains largely unclear how many SPILE genes in the A/C clade were present in the bivalve common ancestor, and which of these genes showed D-quadrant-specific expression. Only the TALE-V clade was supported by high bootstrap values as a clade composed of SPILE genes from distant bivalve species. (Fig. 2, Additional file 1: Figs. S2 and S9–12); however, Cg*TALE6*, one of the genes in the TALE-V clade, showed low expression levels at any developmental stage in oysters (RPKM < 1; [45]), suggesting this gene may not play a role in blastomere fate specification. Future studies should investigate the evolutionary history of SPILE genes in bivalves through further characterisation of SPILE genes from additional bivalve species.

It should be noted that there are phylogenetically related pairs of mussel SPILE genes (Fig. 2 and Additional file 1: Fig. S2), most of which have similar or identical expression patterns. For example, Mv*SPILE-7* and *-8* showed identical expression patterns (Fig. 6s–jj). In addition, both Mv*SPILE-6* and *-10* showed macromere-specific expression at the 16-cell stage, although the bootstrap value supporting the clade was not high (bootstrap values < 50; Fig. 2 and Additional file 1: Fig. S2; Fig. 6e and f, n and o). Furthermore, the closely related Mv*SPILE-4* and *-5* as well as Mv*SPILE-3* in B-clade showed 2d-blastomere-specific expression at the 16-cell stage (Fig. 5b, f, j), suggesting a close linkage of phylogenetic relationships and expression patterns. However, Mv*SPILE-11* and *-12* show complementary rather than similar expression (Fig. 7), implying that significant functional differentiation occurred after gene duplication.

In the classical neofunctionalisation scenario, one gene can acquire a new function after gene duplication because the other gene retains its ancestral function [31]. However, no SPILE genes from the B and A/C clades share identical expression patterns between bivalves and limpets ([29]; Fig. 8). This suggests that, in contrast to classical theory, the expression patterns and functions of several SPILE genes have changed in at least one lineage. In limpets, the SPILE genes participate in specifying the fate of each quartet in early development [29], and there was presumably a similar function in the most common recent ancestor of lophotrochozoans. It may be useful to assess how the expression patterns of genes with important functions have been altered without developmental disruption. It is possible that other transcription factors have similar functions and can compensate for changes in SPILE function. Many lineage-specific homeobox genes are expressed during the early cleavage stages of bivalves [27]. Analysis of the expression patterns and functions of such transcription factors may enable the elucidation of the mechanisms by which changes in the expression patterns of SPILE genes are permitted.

## Conclusion

Our identification of SPILE genes in the transcriptome of *M. virgata* and subsequent phylogenetic analysis of SPILE genes of molluscs suggested that the duplications of SPILE genes had occurred in the bivalve lineage. Among the duplicated SPILE genes, some exhibited lineage-specific expression patterns in the D quadrant or in the C and D quadrants, suggesting that the expansion of bivalve SPILE genes contributed to the evolution of the cell-fate specification patterns in the bivalve lineage. Furthermore, our results imply that the lineage-specific expansion of transcription factors facilitated the evolution of lineage-specific developmental patterns.

## Materials and methods

### Sample acquisition and rearing

Sexually mature individuals of the purplish bifurcate mussel *M. virgata* were collected from Hiraiso, Ibaraki Prefecture (Japan), and Tsuyazaki, Fukuoka Prefecture (Japan) during the breeding season (July to September). In the laboratory, the animals were reared in artificial seawater. Spawning induction and in vitro fertilisation were performed as described previously [46]. After fertilisation, the embryos were reared in artificial seawater at 25 °C.

### RNA sequencing

To collect SPILE sequences from mussels, RNA sequencing (RNA-seq) analysis was performed on samples from multiple early developmental stages of *M. virgata*, including the cleavage, gastrula and trochophore stages (160 min post-fertilisation (mpf), 200 mpf, 235 mpf, 4.5 h post-fertilisation (hpf), 7 hpf and 12.5 hpf). RNA from pooled samples was extracted using the TRIzol reagent (Life Technologies), then treated with DNase and cleaned using an RNeasy kit (Qiagen). Preparation of a paired-end library was performed using the TruSeq Stranded mRNA Sample Preparation Kit (Illumina) and the library was sequenced on a HiSeq2000 platform (Illumina) at the Beijing Genomics Institute. Short reads were deposited in the DNA Data Bank of Japan Sequence Read Archive (DRA011111). Low-quality reads were filtered using the IlluQC_PRLL.pl in the NGSQCToolkit (v2.3) [47] with default parameter settings. De novo assembly was performed using Trinity with default settings (version: trinityrnaseq_r20140717)[48].

### Sequence collection, alignment, and phylogenetic analysis

To identify TALE genes in *M. virgata*, BLAST searches (version 2.2.31 + ; e-value threshold 1e^−5^) were performed using TALE sequences from the Pacific oyster *C. gigas* as the query against the de novo assembly from mussel RNA-seq. The top ten hit sequences of each query were annotated, which revealed 24 TALE genes (Additional file 2: Table S1, 2). The search for open reading frames from identified mussel TALE sequences was conducted using SnapGene Viewer software (Chicago, IL, USA) (http://www.snapgene.com/). The mussel TALE sequences were aligned with TALE sequences from various bilaterians [29, 45, 49–52] using MAFFT [53] with the L-INS-i option, then manually edited using Seaview [54]. Based on the 63 amino acids TALE homeodomain sequences, maximum likelihood trees were inferred using RAxML 8.2.12 [55]. The bootstrap values were calculated using 1000 replicates. Amino acid substitution models were predicted using modelgenerator software [56]. A search for SPILE genes in the A/C clades from chiton, gastropods, and bivalve species was performed by combining BLAST and phylogenetic analyses. A BLAST search was conducted using the full-length (against the gene) or homeodomain (against the genome) sequences of the A/C-clade SPILE genes as a query. We used SPILE A/C-clade genes of *C. gigas* and *M. virgutus* for BLAST searches against bivalve species, and genes of *C. gigas* and *N. fuscoviridis* for BLAST searches against chiton and gastropods. The e-value threshold was set as 1e^−10^, and -seg no option was used. The top five hits (Additional file 2: Table S1) were aligned with the homeodomain sequence of annotated molluscan SPILE genes using MAFFT with the L-INS-i option to identify homeodomain sequences. Phylogenetic analysis using TALE genes from bilaterians and extracted homeodomains were performed to identify the SPILE genes (Additional file 1: Fig. S8). Subsequently, phylogenetic analysis of extracted SPILE genes and other molluscan SPILE genes was performed to identify the SPILE gene in the A/C-clade (Additional file 1: Fig. S9, excluding rapidly evolved SPILE genes; and Additional file 1: Fig. S10; using bivalves SPILE genes alone). Sequences located on different scaffolds but showing high similarity at the nucleotide sequence level (> 97% identity) were considered to be alleles and were excluded from gene counts and analyses (Additional file 2: Table S2). The information of TALE genes used in this study is provided in Additional file 2: Table S2. The alignments of SPILE genes from molluscs for searching conserved sequence features were conducted by MAFFT with the E-INS-i option. Visualisation of alignments was conducted using Seaview [54]. The gene models for mussel TALE genes predicted by the de novo assembly are supplied in Additional file 3: Dataset S1. The TALE sequences used in this study are provided in Additional file 4: Dataset S2, Additional file 5: Dataset S3, Additional file 6: Dataset S4.

### Whole-mount in situ hybridisation

Genes were amplified using the primers listed in Additional file 2: Table S3. RNA probes for in situ hybridisation were prepared according to previously described methods [57]. Embryos for in situ hybridisation were fixed in a solution containing 4% paraformaldehyde, 0.1 M MOPS (pH 7.0) and 0.5 M NaCl. They were then stored in 100% methanol at – 20 °C freezer. Whole-mount in situ hybridisation was performed as described previously [29], although the H_2_O_2_ bleaching procedure was omitted. After in situ hybridisation had been performed, DAPI (SlowFade® Diamond Antifade Mountant with DAPI, Invitrogen/Thermo Fisher Scientific) was added to the samples to aid in blastomere identification. DAPI images for Mv*SPILE-3* to *-12* at 8-cell and 16-cell stages are provided in Additional file 1: Fig. S13. Consistent results were obtained from at least two independent experiments, and at least 10 embryos showed the expression pattern shown in the figures.

## Supplementary Information

**Additional file 1: Fig. S1.** Molecular phylogenetic tree of TALE genes from bilaterians. **Fig. S2.** Molecular phylogenetic tree of SPILE genes from molluscs excluding two rapidly evolving MvSPILE genes. **Fig. S3.** Alignment of SPILE genes in B-clade. **Fig. S4.** Alignment of SPILE genes in D-clade. **Fig. S5.** Conserved region upstream of the homeodomain in A/C-clade SPILE genes. **Fig. S6.** Conserved region upstream of homeodomain in A/C-, B- and D-clade SPILE genes. **Fig. S7.** Alignment of SPILE genes in A/C- and E-clade. **Fig. S8.** Molecular phylogenetic tree of TALE genes from bilaterians and additional molluscan species. **Fig. S9.** Molecular phylogenetic tree of SPILE genes from molluscan species excluding four rapidly evolving SPILE genes. **Fig. S10.** Molecular phylogenetic tree of SPILE genes from bivalves. **Fig. S11.** Molecular phylogenetic tree of SPILE genes from molluscan species excluding four rapidly evolving SPILE genes and B- and E-clade SPILE genes. **Fig. S12.** Molecular phylogenetic tree of SPILE genes from bivalve species excluding four rapidly evolving SPILE genes and B- and E-clade SPILE genes. **Fig. S13.** Expression patterns of SPILE genes of *Mytilisepta virgate*.

**Additional file 2: Table S1.** Results of Blast searches. **Table S2.** Lists and annotations of TALE genes used in this study. **Table S3.** Primers for gene isolation.

**Additional file 3. **Dataset S1 Sequences of TALE genes from mussel transcriptome assembly.

**Additional file 4. **Dataset S2 Sequences of homeodomain (63 amino acids) from bilaterians for phylogenetic analysis of Fig. S1.

**Additional file 5. **Dataset S3 Sequences of SPILE genes in molluscs for Fig. S3-7.

**Additional file 6. **Dataset S4 Sequences of extracted TALE homeodomain (63 amino acids) from molluscan species.

## Data Availability

The data supporting the findings of this study are available within the article and its supplementary materials. The transcriptome assembly of *M. virgata* has been deposited in Figshare (https://doi.org/10.6084/m9.figshare.16565148).

## Abbreviations

TALE: Three-amino-acid loop extension
SPILE: Spiralian-TALE
mpf: Minutes post-fertilisation
hpf: Hours post-fertilisation
