# Supplementary material for: Duplication of spiralian-specific TALE genes and evolution of the blastomere specification mechanism in the bivalve lineage
Source: EvoDevo. 2021 Oct 18;12:11. doi: 10.1186/s13227-021-00181-2 (PMC8524836; doi:10.1186/s13227-021-00181-2)
Supplement: Supplementary file 1 — Additional file 1: Fig. S1. Molecular phylogenetic tree of TALE genes from bilaterians. Fig. S2. Molecular phylogenetic tree of SPILE genes from molluscs excluding two rapidly evolving MvSPILE genes. Fig. S3. Alignment of SPILE genes in B-clade. Fig. S4. Alignment of SPILE genes in D-clade. Fig. S5. Conserved region upstream of the homeodomain in A/C-clade SPILE genes. Fig. S6. Conserved region upstream of homeodomain in A/C-, B- and D-clade SPILE genes. Fig. S7. Alignment of SPILE genes in A/C- and E-clade. Fig. S8. Molecular phylogenetic tree of TALE genes from bilaterians and additional molluscan species. Fig. S9. Molecular phylogenetic tree of SPILE genes from molluscan species excluding four rapidly evolving SPILE genes. Fig. S10. Molecular phylogenetic tree of SPILE genes from bivalves. Fig. S11. Molecular phylogenetic tree of SPILE genes from molluscan species excluding four rapidly evolving SPILE genes and B- and E-clade SPILE genes. Fig. S12. Molecular phylogenetic tree of SPILE genes from bivalve species excluding four rapidly evolving SPILE genes and B- and E-clade SPILE genes. Fig. S13. Expression patterns of SPILE genes of Mytilisepta virgate. [file 13227_2021_181_MOESM1_ESM.pdf]

Additional file1: Additional Figures S1-S13

Duplication of spiralian-specific TALE genes and evolution of the blastomere specification mechanism in the bivalve lineage

Supanat Phuangphong[1]*, Jumpei Tsunoda[1], Hiroshi Wada[2], Yoshiaki Morino[2]*

[1] Graduate School of Life and Environmental Sciences, University of Tsukuba, Tsukuba, Ibaraki, 305-8572, Japan.

[2] Faculty of Life and Environmental Sciences, University of Tsukuba, Tsukuba, Ibaraki, 305-8572, Japan.

*Correspondence

supanat.phuangphong@gmail.com

morino.yoshiaki.ge@u.tsukuba.ac.jp

List of Additional Figures

Fig. S1 Molecular phylogenetic tree of TALE genes from bilaterians

Fig. S2 Molecular phylogenetic tree of SPILE genes from molluscs excluding two rapidly evolving MvSPILE genes.

Fig. S3 Alignment of SPILE genes in B-clade.

Fig. S4 Alignment of SPILE genes in D-clade.

Fig. S5 Conserved region upstream of the homeodomain in A/C-clade SPILE genes

Fig. S6 Conserved region upstream of homeodomain in A/C-, B- and D-clade SPILE genes

Fig. S7 Alignment of SPILE genes in A/C- and E-clade.

Fig. S8 Molecular phylogenetic tree of TALE genes from bilaterians and additional molluscan species.

Fig. S9 Molecular phylogenetic tree of SPILE genes from molluscan species excluding four rapidly evolving SPILE genes.

Fig. S10 Molecular phylogenetic tree of SPILE genes from bivalves.

Fig. S11 Molecular phylogenetic tree of SPILE genes from molluscan species excluding four rapidly evolving SPILE genes and B- and E-clade SPILE genes.

Fig. S12 Molecular phylogenetic tree of SPILE genes from bivalve species excluding four rapidly evolving SPILE genes and B- and E-clade SPILE genes.

Fig. S13 Expression patterns of SPILE genes of *Mytilisepta virgata*

Fig. S1

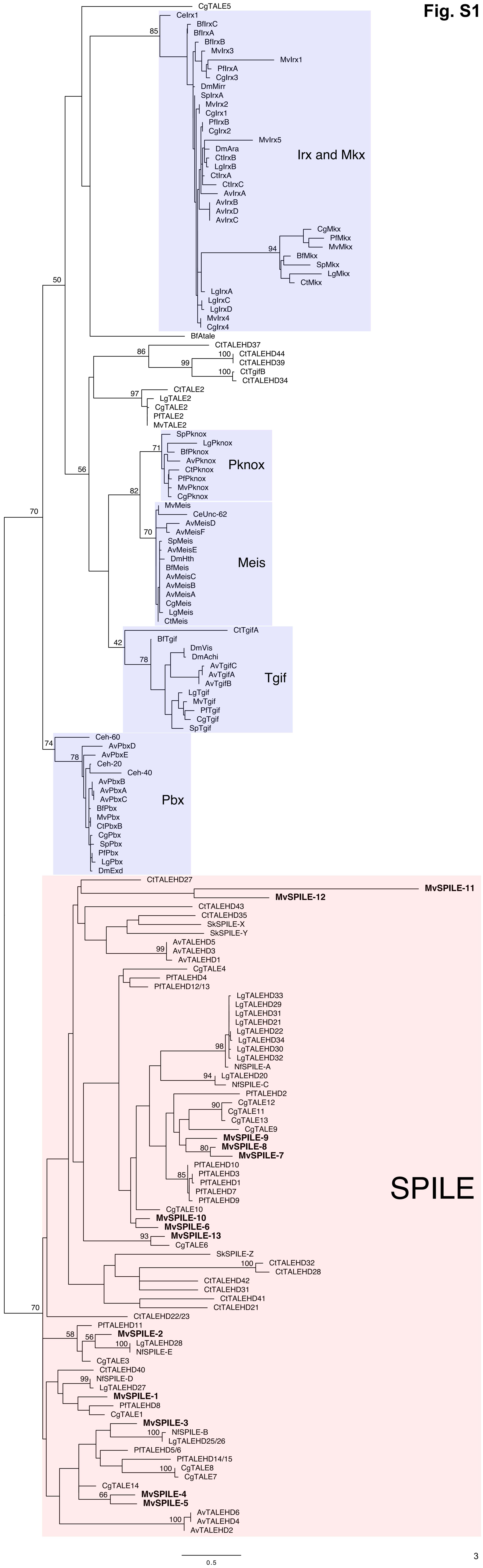

CgTALE5
CeIrx1
85
BfIrxC
BfIrxA
BfIrxB
MvIrx3
MvIrx1
PfIrxA
CgIrx3
DmMirr
SpIrxA
MvIrx2
CgIrx1
PfIrxB
CgIrx2
MvIrx5
DmAra
CtIrxB
LgIrxB
CtIrxA
CtIrxC
AvIrxA
AvIrxB
AvIrxD
AvIrxC
Irx and Mkx
CgMkx
PfMkx
MvMkx
94
BfMkx
SpMkx
LgMkx
CtMkx
LgIrxA
LgIrxC
LgIrxD
MvIrx4
CgIrx4
50
BfAtale
CtTALEHD37
86
100
CtTALEHD44
CtTALEHD39
99
100
CtTgifB
CtTALEHD34
CtTALE2
97
LgTALE2
CgTALE2
PfTALE2
MvTALE2
SpPknox
LgPknox
71
BfPknox
AvPknox
CtPknox
Pknox
PfPknox
MvPknox
CgPknox
56
82
MvMeis
CeUnc-62
AvMeisD
70
AvMeisF
SpMeis
AvMeisE
DmHth
Meis
BfMeis
AvMeisC
AvMeisB
AvMeisA
CgMeis
LgMeis
CtMeis
70
CtTgifA
42
BfTgif
DmVis
78
DmAchi
AvTgifC
AvTgifA
Tgif
AvTgifB
LgTgif
MvTgif
PfTgif
CgTgif
SpTgif
Ceh-60
74
AvPbxD
78
AvPbxE
Ceh-20
Ceh-40
AvPbxB
AvPbxA
AvPbxC
Pbx
BfPbx
MvPbx
CtPbxB
CgPbx
SpPbx
PfPbx
LgPbx
DmExd
CtTALEHD27
MvSPILE-11
MvSPILE-12
CtTALEHD43
CtTALEHD35
SkSPILE-X
SkSPILE-Y
AvTALEHD5
99
AvTALEHD3
AvTALEHD1
CgTALE4
PfTALEHD4
PfTALEHD12/13
LgTALEHD33
LgTALEHD29
LgTALEHD31
LgTALEHD21
LgTALEHD22
LgTALEHD34
98
LgTALEHD30
LgTALEHD32
NfSPILE-A
94
LgTALEHD20
NfSPILE-C
PfTALEHD2
90
CgTALE12
CgTALE11
CgTALE13
CgTALE9
MvSPILE-9
80
MvSPILE-8
MvSPILE-7
PfTALEHD10
85
PfTALEHD3
PfTALEHD1
PfTALEHD7
PfTALEHD9
SPILE
CgTALE10
MvSPILE-10
MvSPILE-6
93
MvSPILE-13
CgTALE6
SkSPILE-Z
100
CtTALEHD32
CtTALEHD28
CtTALEHD42
CtTALEHD31
CtTALEHD41
CtTALEHD21
70
CtTALEHD22/23
PfTALEHD11
58
56
MvSPILE-2
100
LgTALEHD28
NfSPILE-E
CgTALE3
CtTALEHD40
99
NfSPILE-D
LgTALEHD27
MvSPILE-1
PfTALEHD8
CgTALE1
MvSPILE-3
100
NfSPILE-B
LgTALEHD25/26
PfTALEHD5/6
PfTALEHD14/15
100
CgTALE8
CgTALE7
CgTALE14
66
MvSPILE-4
MvSPILE-5
100
AvTALEHD6
AvTALEHD4
AvTALEHD2
0.5

Fig. S1 Molecular phylogenetic tree of the TALE genes from bilaterians

This tree was constructed based on the amino acid sequences of the homeodomains using the maximum likelihood (ML) method. The amino acid substitution model was LG + G. The numbers at the nodes are the bootstrap values (only those ≥ 50% or selected are shown). Dm: *Drosophila melanogaster*, Ce: *Caenorhabditis elegans,* Bf: *Branchiostoma floridae*, Sp: *Strongylocentrotus purpuratus,* Cg: *Crassostrea gigas*, Pf: *Pinctada fucata*, Lg: *Lottia gigantea*, Ct: *Capitella teleta*, Av*: Adenita vaga*, Nf: *Nipponacmea fuscoviridis,* Sk: *Spirobranchus kraussii*. Mv*: Mytilisepta virgata*

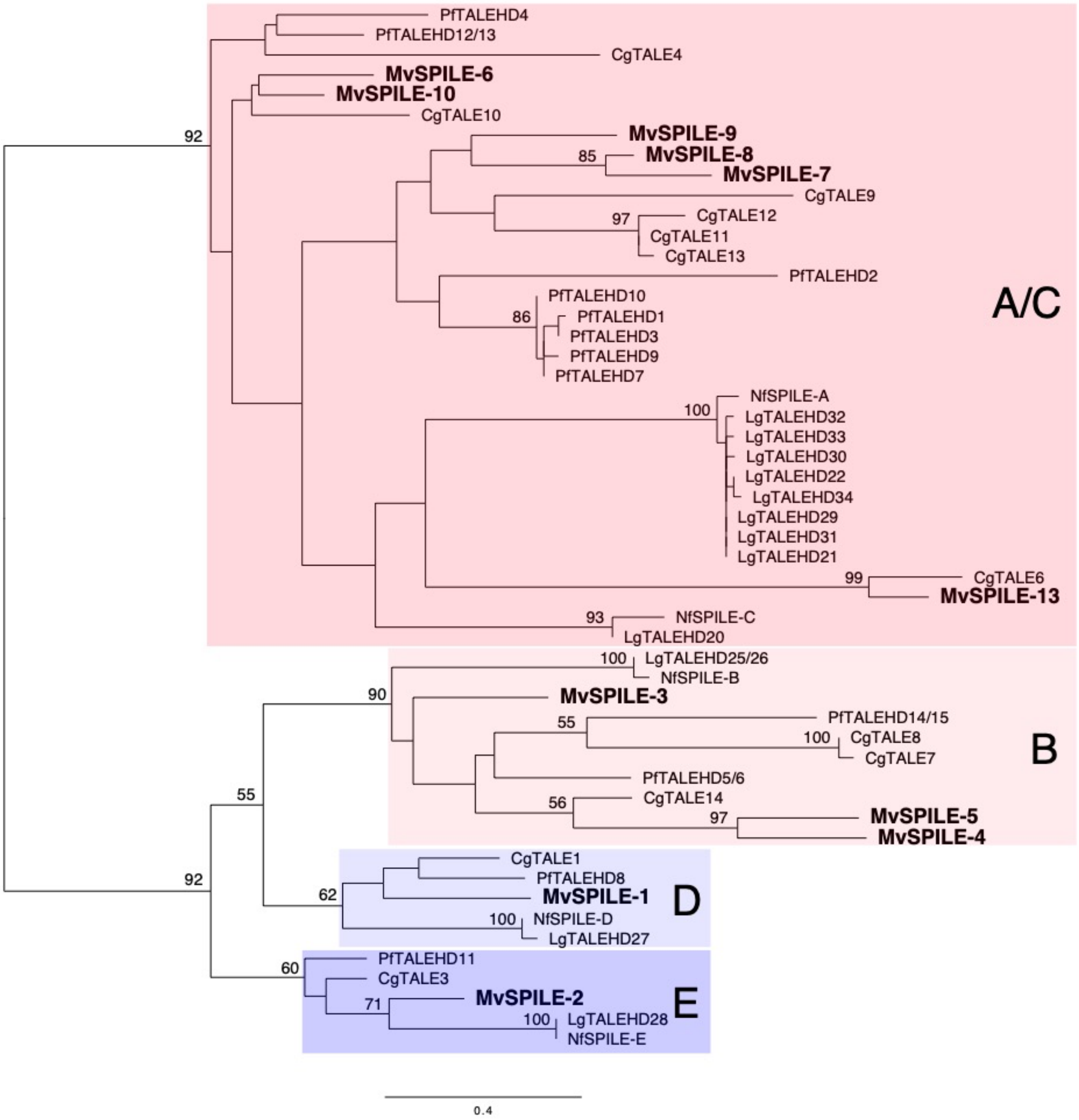

Fig. S2 Molecular phylogenetic tree of the SPILE genes from mollusc excluding two rapidly evolving MvSPILE genes

Molecular phylogenetic tree of the SPILE genes from mollusc excluding two rapidly evolving MvSPILE genes, Mv*SPILE-11* and Mv*SPILE-12*. Tree was constructed based on the amino acid sequences of the homeodomains using the maximum likelihood (ML) method. The amino acid substitution model was LG + G + F. The numbers at the nodes are the bootstrap values (only those ≥ 50% and selected are shown). Cg: *Crassostrea gigas*, Pf: *Pinctada fucata*, Lg: *Lottia gigantea*, Nf: *Nipponacmea fuscoviridis,* Mv*: Mytilisepta virgata*

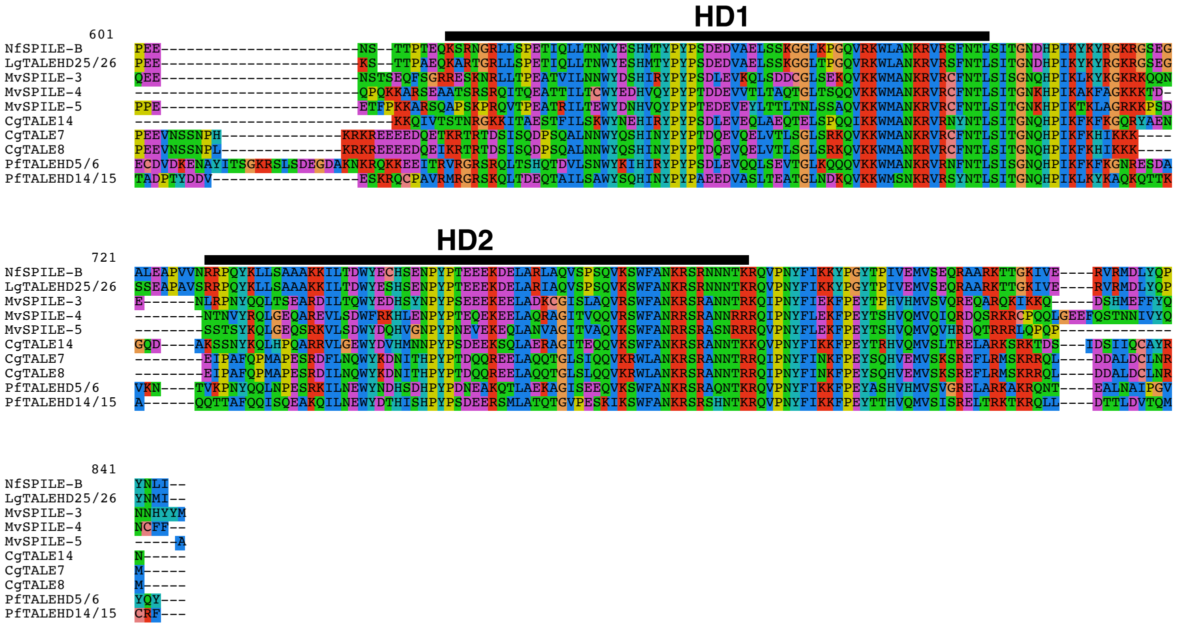

Fig. S3 Alignment of SPILE genes in B-clade.

Amino acid sequences of SPILE-B clades genes are aligned by mafft with E-INS-i option. The region around the homeodomain is shown. HD1 and HD2 indicate the position of homeodomain 1 and 2, respectively.

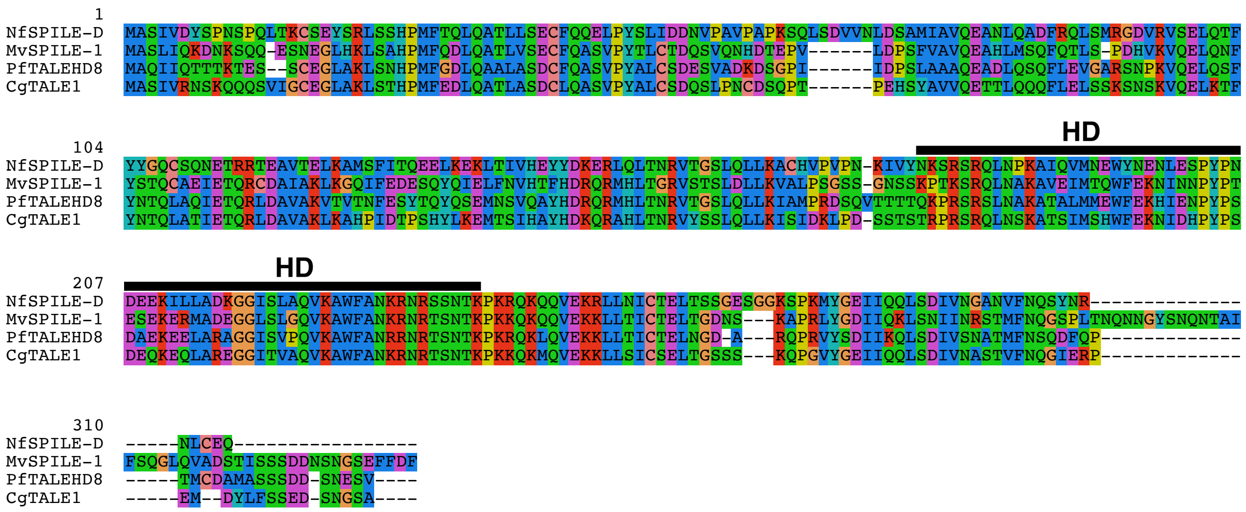

Fig. S4 Alignment of SPILE genes in D-clade.

Amino acid sequences of SPILE-D clades genes are aligned by mafft with L-INS-i option.

HD indicates the position of homeodomain. The sequences outside the homeodomain were also similar among the D-clade genes.

Fig. S5

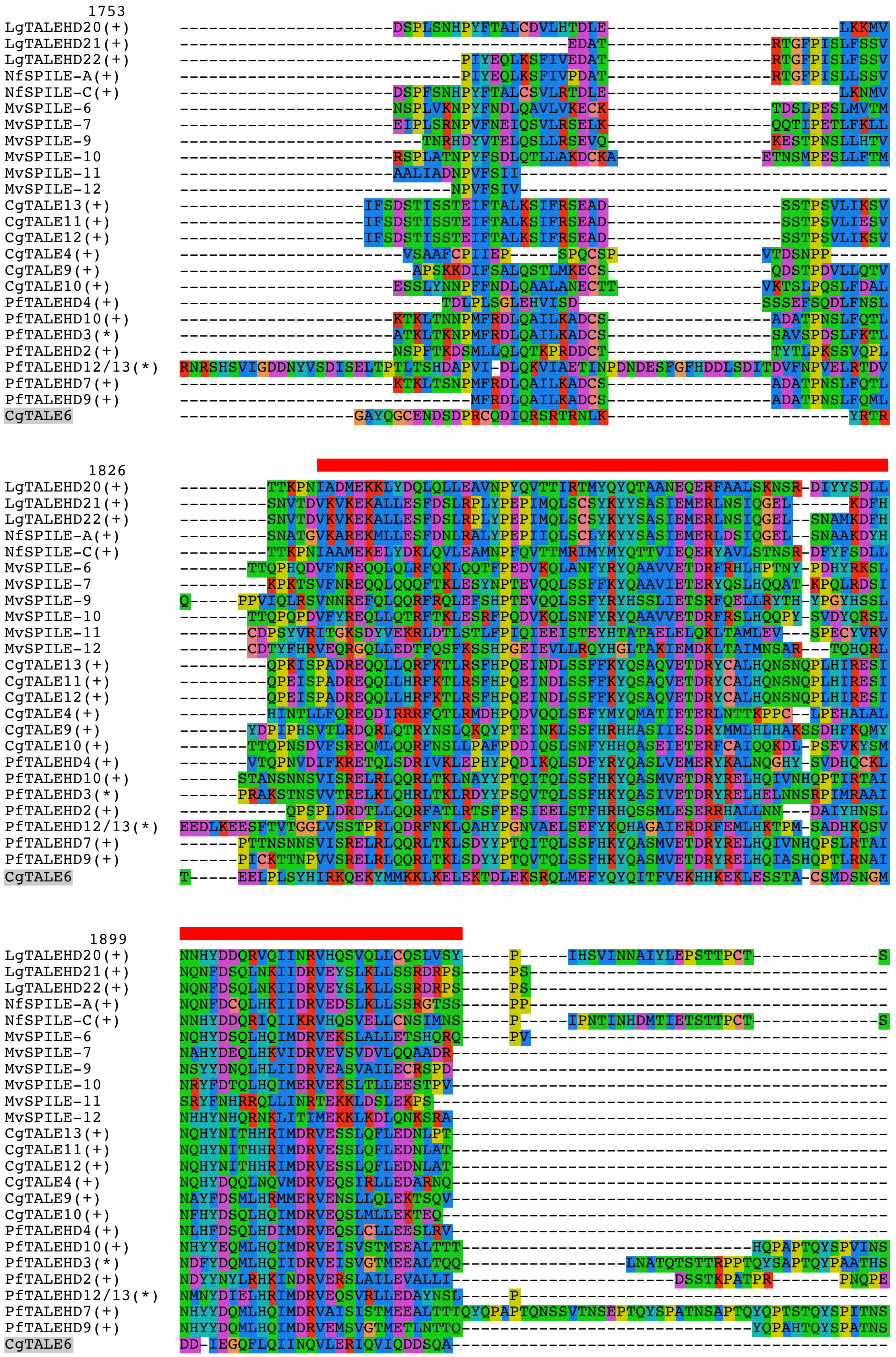
1753
1826
1899
LgTALEHD20(+)
LgTALEHD21(+)
LgTALEHD22(+)
NfSPILE-A(+)
NfSPILE-C(+)
MvSPILE-6
MvSPILE-7
MvSPILE-9
MvSPILE-10
MvSPILE-11
MvSPILE-12
CgTALE13(+)
CgTALE11(+)
CgTALE12(+)
CgTALE4(+)
CgTALE9(+)
CgTALE10(+)
PfTALEHD4(+)
PfTALEHD10(+)
PfTALEHD3(*)
PfTALEHD2(+)
PfTALEHD12/13(*)
PfTALEHD7(+)
PfTALEHD9(+)
CgTALE6

Fig. S5 Conserved region upstream of the homeodomain in A/C-clade SPILE genes

Amino acid sequences of SPILE A/C-clades genes are aligned by mafft with E-INS-i option. The regions around the conserved sequences are shown. Red bars indicate region of PADRE domain. Plus (+) indicates genes that Barton-Owen et al. (2018) suggested have a PADRE domain, and asterisks (*) indicate that the gene model used in Barton-Owen et al. (2018) was fragmentary and did not contain the region corresponding to the PADRE domain. Fragmented genes (Mv*SPILE-8*, Mv*SPILE-13*, and Pf*TALEHD1*) are not included. The Cg*TALE6* (grey) sequence is aligned, but is less conserved, suggesting the absence of a PADRE domain.

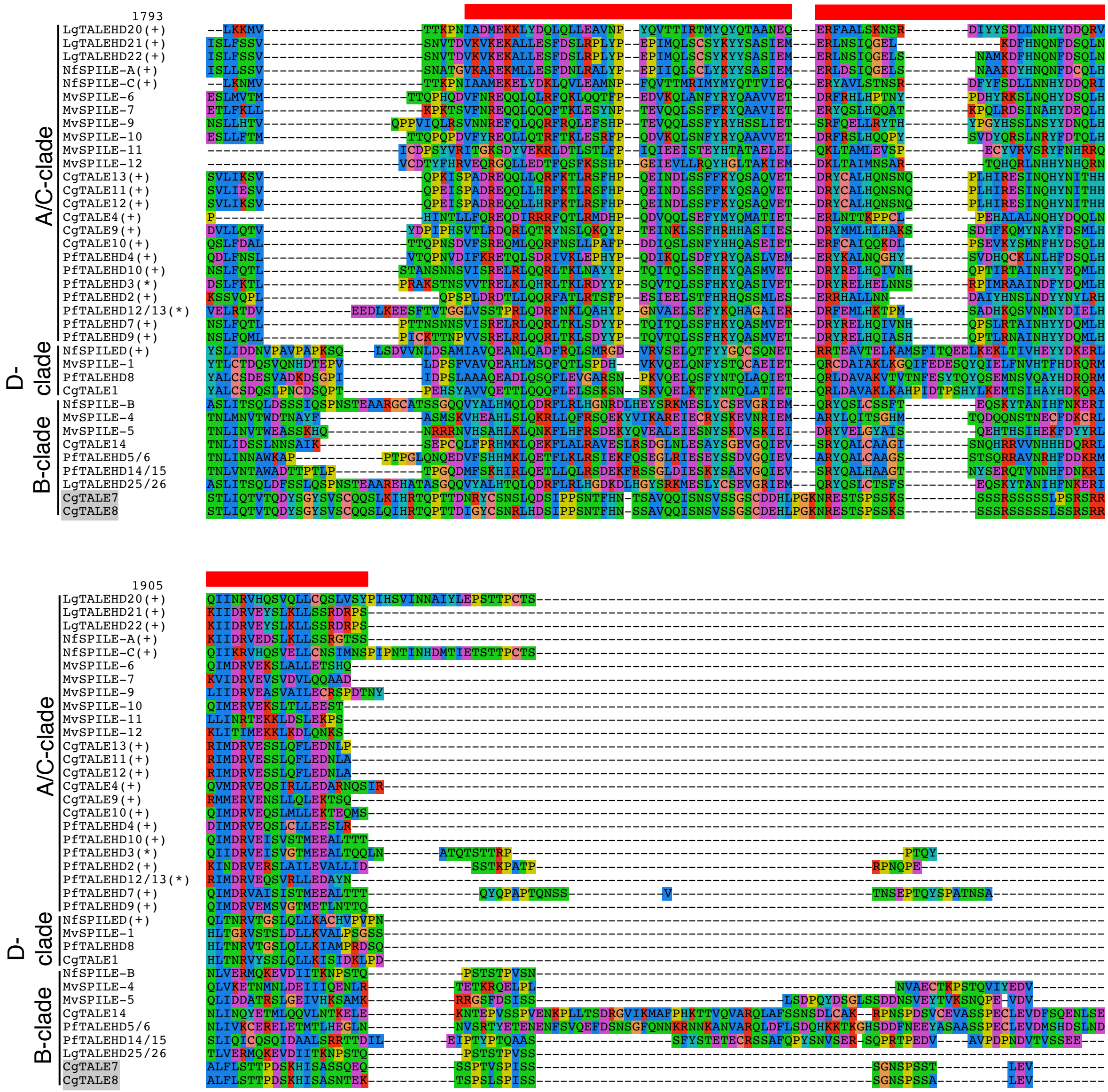

Fig. S6 Conserved region upstream of the homeodomain in A/C-, B- and D-clade SPILE genes

Amino acid sequences of A/C-, B- and D-clades SPILE genes are aligned by mafft with E-INS-i option. The regions around conserved sequences are shown. Red bars indicate region of PADRE domain. Plus (+) indicates genes that Barton-Owen et al. (2018) suggested have a PADRE domain, and asterisks (*) indicate that the gene model used in Barton-Owen et al. (2018) was fragmentary and did not contain the region corresponding to the PADRE domain. Fragmented genes (Mv*SPILE-8*, Mv*SPILE-13*, and Pf*TALEHD1*) and Cg*TALE6* are not included. The Cg*TALE7* and *-8* (grey) sequences are aligned, but are less conserved, suggesting the absence of a PADRE domain.

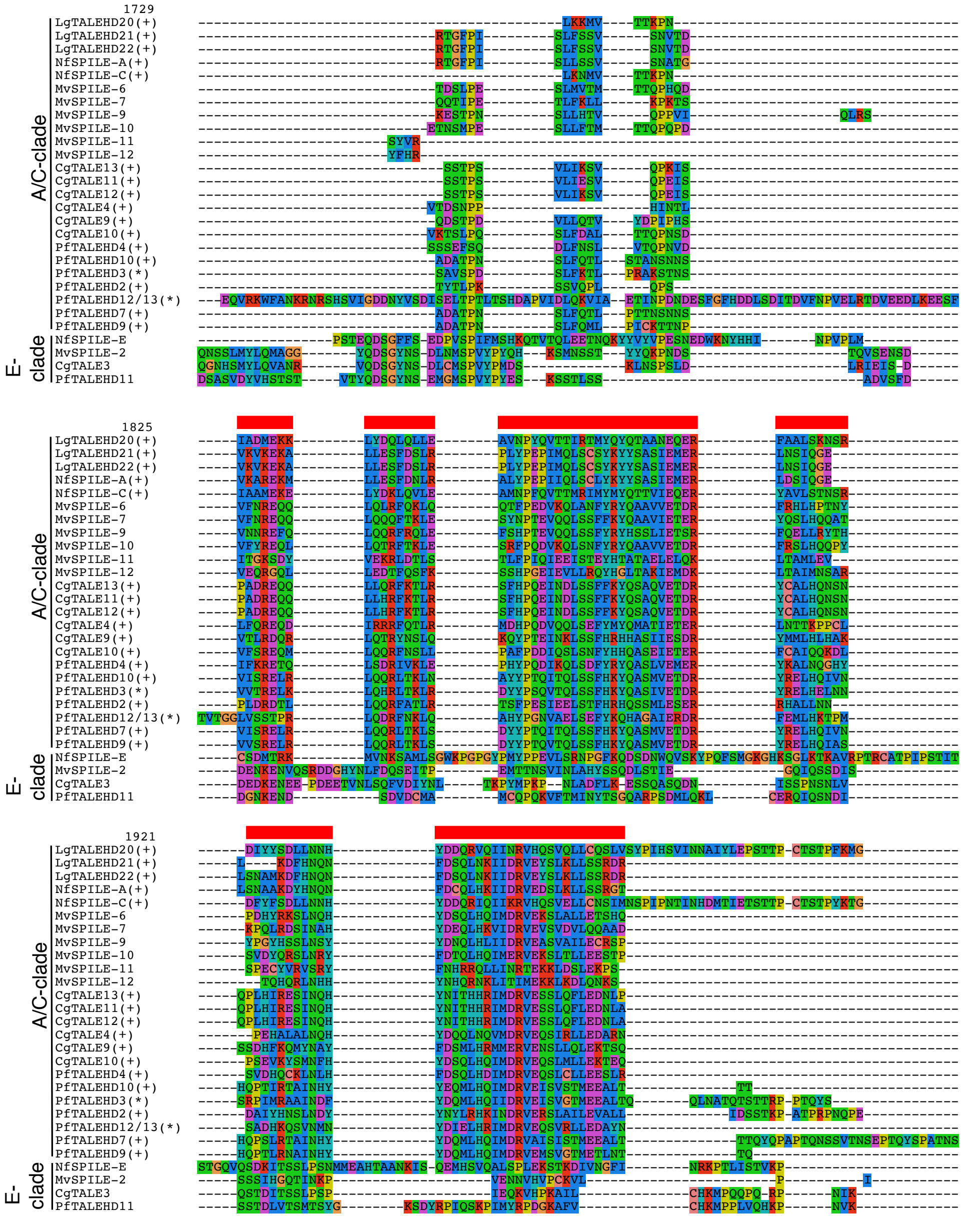

Fig. S7 Alignment of SPILE genes in A/C- and E-clade.

Amino acid sequences of A/C- and E- clades SPILE genes are aligned by mafft with E-INS-i option. The regions around PADRE domains are shown. Red bars indicate the region of PADRE domain. The amino acid sequences of E-clade SPILE genes are poorly aligned with PADRE domains, suggesting the absence of a PADRE domain. Fragmented genes (Mv*SPILE-8*, Mv*SPILE-13*, and Pf*TALEHD1*) and Cg*TALE6* are not included.

Fig. S8

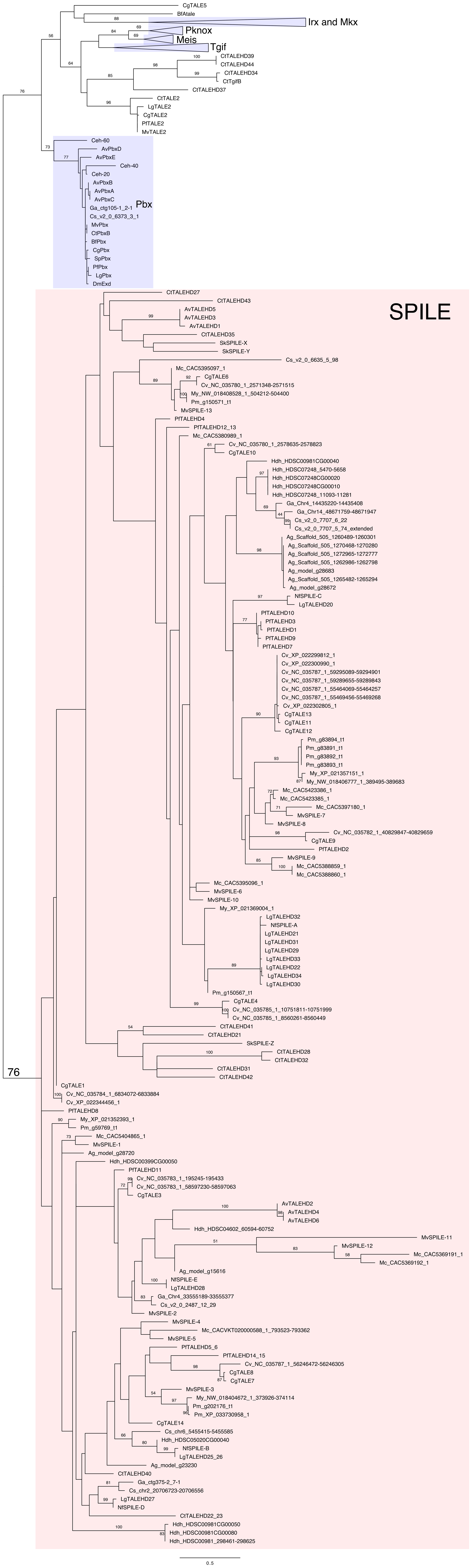

CgTALE5
BfAtale
Irx and Mkx
Pknox
Meis
Tgif
CtTALEHD39
CtTALEHD44
CtTALEHD34
CtTgifB
CtTALEHD37
CtTALE2
LgTALE2
CgTALE2
PfTALE2
MvTALE2
Ceh-60
AvPbxD
AvPbxE
Ceh-40
Ceh-20
AvPbxB
AvPbxA
AvPbxC
Ga_ctg105-1_2-1
Cs_v2_0_6373_3_1
Pbx
MvPbx
CtPbxB
BfPbx
CgPbx
SpPbx
PfPbx
LgPbx
DmExd
SPILE
CtTALEHD27
CtTALEHD43
AvTALEHD5
AvTALEHD3
AvTALEHD1
CtTALEHD35
SkSPILE-X
SkSPILE-Y
Cs_v2_0_6635_5_98
Mc_CAC5395097_1
CgTALE6
Cv_NC_035780_1_2571348-2571515
My_NW_018408528_1_504212-504400
Pm_g150571_t1
MvSPILE-13
PfTALEHD4
PfTALEHD12_13
Mc_CAC5380989_1
Cv_NC_035780_1_2578635-2578823
CgTALE10
Hdh_HDSC00981CG00040
Hdh_HDSC07248_5470-5658
Hdh_HDSC07248CG00020
Hdh_HDSC07248CG00010
Hdh_HDSC07248_11093-11281
Ga_Chr4_14435220-14435408
Ga_Chr14_48671759-48671947
Cs_v2_0_7707_6_22
Cs_v2_0_7707_5_74_extended
Ag_Scaffold_505_1260489-1260301
Ag_Scaffold_505_1270468-1270280
Ag_Scaffold_505_1272965-1272777
Ag_Scaffold_505_1262986-1262798
Ag_model_g28683
Ag_Scaffold_505_1265482-1265294
Ag_model_g28672
NfSPILE-C
LgTALEHD20
PfTALEHD10
PfTALEHD3
PfTALEHD1
PfTALEHD9
PfTALEHD7
Cv_XP_022299812_1
Cv_XP_022300990_1
Cv_NC_035787_1_59295089-59294901
Cv_NC_035787_1_59289655-59289843
Cv_NC_035787_1_55464069-55464257
Cv_NC_035787_1_55469456-55469268
Cv_XP_022302805_1
CgTALE13
CgTALE11
CgTALE12
Pm_g83894_t1
Pm_g83891_t1
Pm_g83892_t1
Pm_g83893_t1
My_XP_021357151_1
My_NW_018406777_1_389495-389683
Mc_CAC5423386_1
Mc_CAC5423385_1
Mc_CAC5397180_1
MvSPILE-7
MvSPILE-8
Cv_NC_035782_1_40829847-40829659
CgTALE9
PfTALEHD2
MvSPILE-9
Mc_CAC5388859_1
Mc_CAC5388860_1
Mc_CAC5395096_1
MvSPILE-6
MvSPILE-10
My_XP_021369004_1
LgTALEHD32
NfSPILE-A
LgTALEHD21
LgTALEHD31
LgTALEHD29
LgTALEHD33
LgTALEHD22
LgTALEHD34
LgTALEHD30
Pm_g150567_t1
CgTALE4
Cv_NC_035785_1_10751811-10751999
Cv_NC_035785_1_8560261-8560449
CtTALEHD41
CtTALEHD21
SkSPILE-Z
CtTALEHD28
CtTALEHD32
CtTALEHD31
CtTALEHD42
76
CgTALE1
Cv_NC_035784_1_6834072-6833884
Cv_XP_022344456_1
PfTALEHD8
My_XP_021352393_1
Pm_g59769_t1
Mc_CAC5404865_1
MvSPILE-1
Ag_model_g28720
Hdh_HDSC00399CG00050
PfTALEHD11
Cv_NC_035783_1_195245-195433
Cv_NC_035783_1_58597230-58597063
CgTALE3
AvTALEHD2
AvTALEHD4
AvTALEHD6
Hdh_HDSC04602_60594-60752
MvSPILE-11
MvSPILE-12
Mc_CAC5369191_1
Mc_CAC5369192_1
Ag_model_g15616
NfSPILE-E
LgTALEHD28
Ga_Chr4_33555189-33555377
Cs_v2_0_2487_12_29
MvSPILE-2
MvSPILE-4
Mc_CACVKT020000588_1_793523-793362
MvSPILE-5
PfTALEHD5_6
PfTALEHD14_15
Cv_NC_035787_1_56246472-56246305
CgTALE8
CgTALE7
MvSPILE-3
My_NW_018404672_1_373926-374114
Pm_g202176_t1
Pm_XP_033730958_1
CgTALE14
Cs_chr6_5455415-5455585
Hdh_HDSC05020CG00040
NfSPILE-B
LgTALEHD25_26
Ag_model_g23230
CtTALEHD40
Ga_ctg375-2_7-1
Cs_chr2_20706723-20706556
LgTALEHD27
NfSPILE-D
CtTALEHD22_23
Hdh_HDSC00981CG00050
Hdh_HDSC00981CG00080
Hdh_HDSC00981_298461-298625
0.5

Fig. S8 Molecular phylogenetic tree of TALE genes from bilaterians additional molluscan species.

Tree was constructed based on the amino acid sequences of the homeodomains using the maximum likelihood (ML) method. The amino acid substitution model was LG + G. The numbers at the nodes are the bootstrap values (only those ≥ 50% and selected are shown). Dm: *Drosophila melanogaster*, Ce: *Caenorhabditis elegans,* Bf: *Branchiostoma floridae*, Sp: *Strongylocentrotus purpuratus,* Cg: *Crassostrea gigas*, Pf: *Pinctada fucata*, Lg: *Lottia gigantea*, Ct: *Capitella teleta*, Av*: Adenita vaga*, Nf: *Nipponacmea fuscoviridis,* Sk: *Spirobranchus kraussii*. Mv*: Mytilisepta virgata*. My: *Mizuhopecten yessoensis*, Pm: *Pecten maximus*, Cv: *Crassostrea virginica*, Mc: *Mytilus coruscus,* Hdh: *Haliotis discus hannai*, Ga: *Gigantopelta aegis*, Cs: *Chrysomallon squamiferum*, Ag: *Acanthopleura granulate*.

Fig. S9

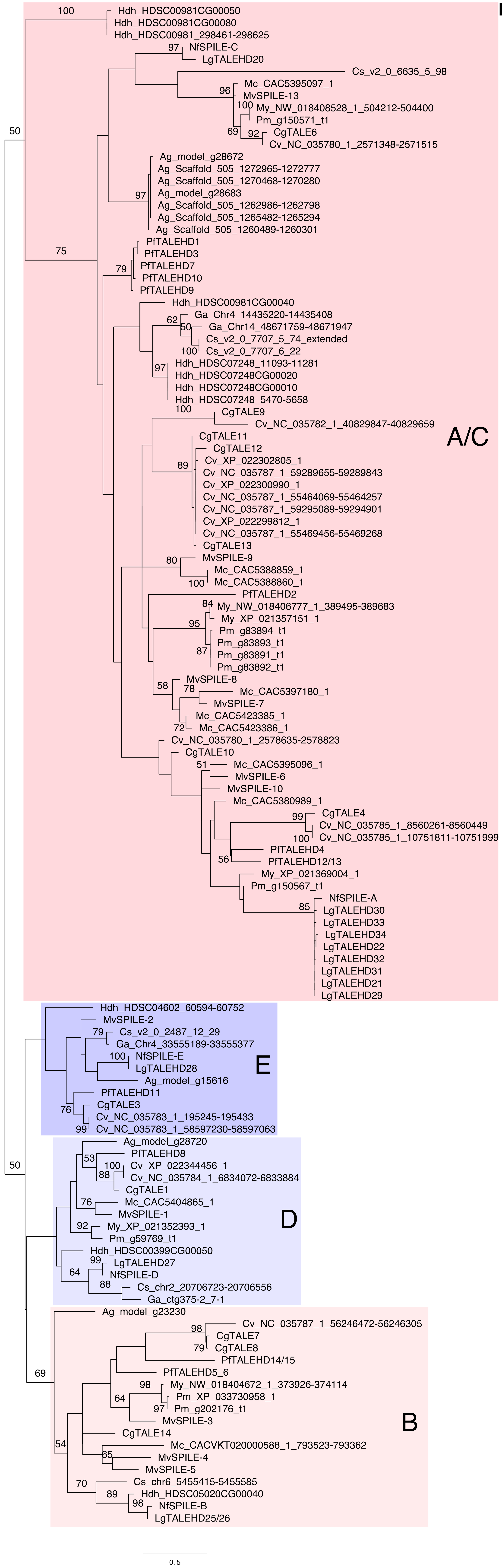
100
Hdh_HDSC00981CG00050
Hdh_HDSC00981CG00080
Hdh_HDSC00981_298461-298625
97
NfSPILE-C
LgTALEHD20
Cs_v2_0_6635_5_98
96
Mc_CAC5395097_1
MvSPILE-13
100
My_NW_018408528_1_504212-504400
Pm_g150571_t1
69
92
CgTALE6
Cv_NC_035780_1_2571348-2571515
50
Ag_model_g28672
Ag_Scaffold_505_1272965-1272777
Ag_Scaffold_505_1270468-1270280
97
Ag_model_g28683
Ag_Scaffold_505_1262986-1262798
Ag_Scaffold_505_1265482-1265294
Ag_Scaffold_505_1260489-1260301
PfTALEHD1
75
PfTALEHD3
79
PfTALEHD7
PfTALEHD10
PfTALEHD9
Hdh_HDSC00981CG00040
62
Ga_Chr4_14435220-14435408
50
Ga_Chr14_48671759-48671947
Cs_v2_0_7707_5_74_extended
100
Cs_v2_0_7707_6_22
97
Hdh_HDSC07248_11093-11281
Hdh_HDSC07248CG00020
Hdh_HDSC07248CG00010
Hdh_HDSC07248_5470-5658
100
CgTALE9
Cv_NC_035782_1_40829847-40829659
A/C
CgTALE11
CgTALE12
89
Cv_XP_022302805_1
Cv_NC_035787_1_59289655-59289843
Cv_XP_022300990_1
Cv_NC_035787_1_55464069-55464257
Cv_NC_035787_1_59295089-59294901
Cv_XP_022299812_1
Cv_NC_035787_1_55469456-55469268
CgTALE13
80
MvSPILE-9
Mc_CAC5388859_1
100
Mc_CAC5388860_1
PfTALEHD2
84
My_NW_018406777_1_389495-389683
95
My_XP_021357151_1
Pm_g83894_t1
Pm_g83893_t1
87
Pm_g83891_t1
Pm_g83892_t1
MvSPILE-8
58
78
Mc_CAC5397180_1
MvSPILE-7
Mc_CAC5423385_1
72
Mc_CAC5423386_1
Cv_NC_035780_1_2578635-2578823
CgTALE10
51
Mc_CAC5395096_1
MvSPILE-6
MvSPILE-10
Mc_CAC5380989_1
99
CgTALE4
Cv_NC_035785_1_8560261-8560449
100
Cv_NC_035785_1_10751811-10751999
PfTALEHD4
56
PfTALEHD12/13
My_XP_021369004_1
Pm_g150567_t1
NfSPILE-A
85
LgTALEHD30
LgTALEHD33
LgTALEHD34
LgTALEHD22
LgTALEHD32
LgTALEHD31
LgTALEHD21
LgTALEHD29
Hdh_HDSC04602_60594-60752
MvSPILE-2
79
Cs_v2_0_2487_12_29
Ga_Chr4_33555189-33555377
100
NfSPILE-E
LgTALEHD28
E
Ag_model_g15616
PfTALEHD11
76
CgTALE3
Cv_NC_035783_1_195245-195433
99
Cv_NC_035783_1_58597230-58597063
Ag_model_g28720
53
PfTALEHD8
100
Cv_XP_022344456_1
50
88
Cv_NC_035784_1_6834072-6833884
CgTALE1
76
Mc_CAC5404865_1
MvSPILE-1
D
92
My_XP_021352393_1
Pm_g59769_t1
Hdh_HDSC00399CG00050
99
LgTALEHD27
64
NfSPILE-D
88
Cs_chr2_20706723-20706556
Ga_ctg375-2_7-1
Ag_model_g23230
98
Cv_NC_035787_1_56246472-56246305
CgTALE7
79
CgTALE8
PfTALEHD14/15
PfTALEHD5_6
69
98
My_NW_018404672_1_373926-374114
64
Pm_XP_033730958_1
97
Pm_g202176_t1
MvSPILE-3
B
CgTALE14
54
Mc_CACVKT020000588_1_793523-793362
65
MvSPILE-4
MvSPILE-5
70
Cs_chr6_5455415-5455585
89
Hdh_HDSC05020CG00040
98
NfSPILE-B
LgTALEHD25/26
0.5

Fig. S9 Molecular phylogenetic tree of SPILE genes from molluscan species excluding four rapidly evolving SPILE genes.

Tree was constructed based on the amino acid sequences of the homeodomains using the maximum likelihood (ML) method. Four rapidly evolving mussel SPILE genes (Mv*SPILE-11*, *-12*, Mc_*CAC5369191*, and Mc_*CAC5369192*) are excluded from analysis. The amino acid substitution model was LG + G. The numbers at the nodes are the bootstrap values (only those ≥ 50% and selected are shown). Cg: *Crassostrea gigas*, Pf: *Pinctada fucata*, Lg: *Lottia gigantea*, Nf: *Nipponacmea fuscoviridis,* Mv*: Mytilisepta virgata*. My: *Mizuhopecten yessoensis*, Pm: *Pecten maximus*, Cv: *Crassostrea virginica*, Mc: *Mytilus coruscus,* Hdh: *Haliotis discus hannai*, Ga: *Gigantopelta aegis*, Cs: *Chrysomallon squamiferum*, Ag: *Acanthopleura granulate*.

Fig. S10

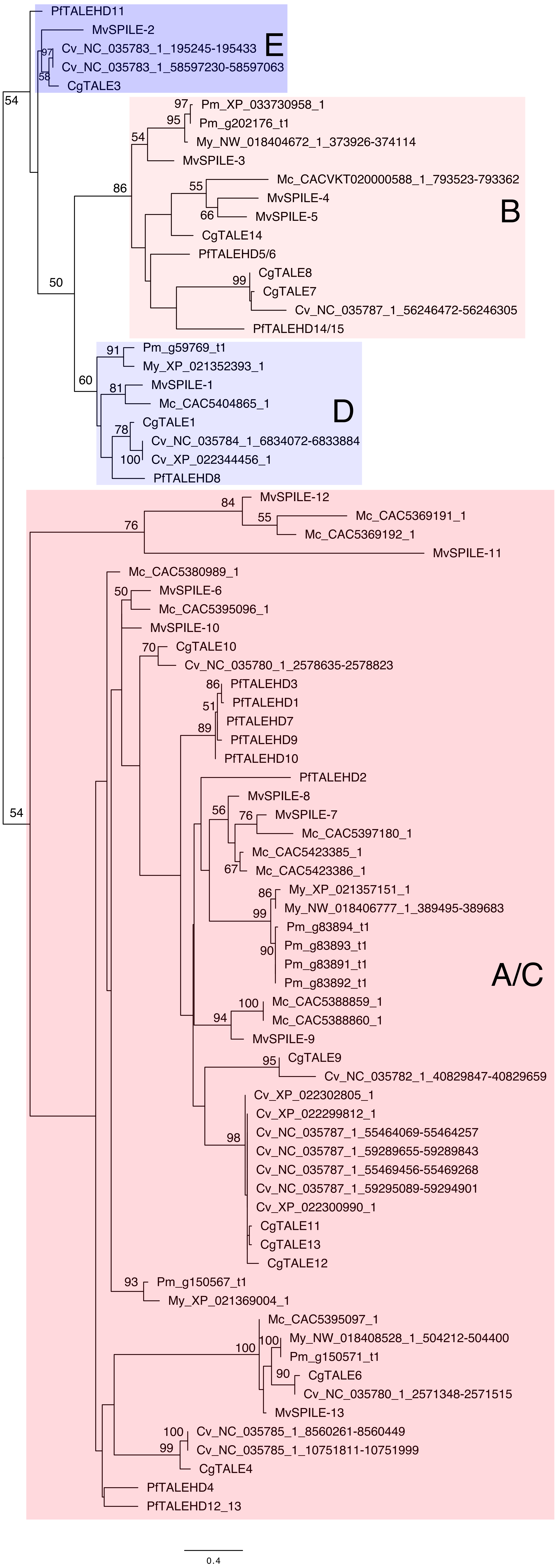

E
PfTALEHD11
MvSPILE-2
Cv_NC_035783_1_195245-195433
Cv_NC_035783_1_58597230-58597063
CgTALE3
B
Pm_XP_033730958_1
Pm_g202176_t1
My_NW_018404672_1_373926-374114
MvSPILE-3
Mc_CACVKT020000588_1_793523-793362
MvSPILE-4
MvSPILE-5
CgTALE14
PfTALEHD5/6
CgTALE8
CgTALE7
Cv_NC_035787_1_56246472-56246305
PfTALEHD14/15
D
Pm_g59769_t1
My_XP_021352393_1
MvSPILE-1
Mc_CAC5404865_1
CgTALE1
Cv_NC_035784_1_6834072-6833884
Cv_XP_022344456_1
PfTALEHD8
A/C
MvSPILE-12
Mc_CAC5369191_1
Mc_CAC5369192_1
MvSPILE-11
Mc_CAC5380989_1
MvSPILE-6
Mc_CAC5395096_1
MvSPILE-10
CgTALE10
Cv_NC_035780_1_2578635-2578823
PfTALEHD3
PfTALEHD1
PfTALEHD7
PfTALEHD9
PfTALEHD10
PfTALEHD2
MvSPILE-8
MvSPILE-7
Mc_CAC5397180_1
Mc_CAC5423385_1
Mc_CAC5423386_1
My_XP_021357151_1
My_NW_018406777_1_389495-389683
Pm_g83894_t1
Pm_g83893_t1
Pm_g83891_t1
Pm_g83892_t1
Mc_CAC5388859_1
Mc_CAC5388860_1
MvSPILE-9
CgTALE9
Cv_NC_035782_1_40829847-40829659
Cv_XP_022302805_1
Cv_XP_022299812_1
Cv_NC_035787_1_55464069-55464257
Cv_NC_035787_1_59289655-59289843
Cv_NC_035787_1_55469456-55469268
Cv_NC_035787_1_59295089-59294901
Cv_XP_022300990_1
CgTALE11
CgTALE13
CgTALE12
Pm_g150567_t1
My_XP_021369004_1
Mc_CAC5395097_1
My_NW_018408528_1_504212-504400
Pm_g150571_t1
CgTALE6
Cv_NC_035780_1_2571348-2571515
MvSPILE-13
Cv_NC_035785_1_8560261-8560449
Cv_NC_035785_1_10751811-10751999
CgTALE4
PfTALEHD4
PfTALEHD12_13
0.4

Fig. S10 Molecular phylogenetic tree of SPILE genes from bivalves.

Tree was constructed based on the amino acid sequences of the homeodomains of bivalve SPILE genes using the maximum likelihood (ML) method. The amino acid substitution model was LG + G. The numbers at the nodes are the bootstrap values (only those ≥ 50% and selected are shown). Cg: *Crassostrea gigas*, Pf: *Pinctada fucata*, Mv*: Mytilisepta virgata*. My: *Mizuhopecten yessoensis*, Pm: *Pecten maximus*, Cv: *Crassostrea virginica*, Mc: *Mytilus coruscus,*

Fig. S11

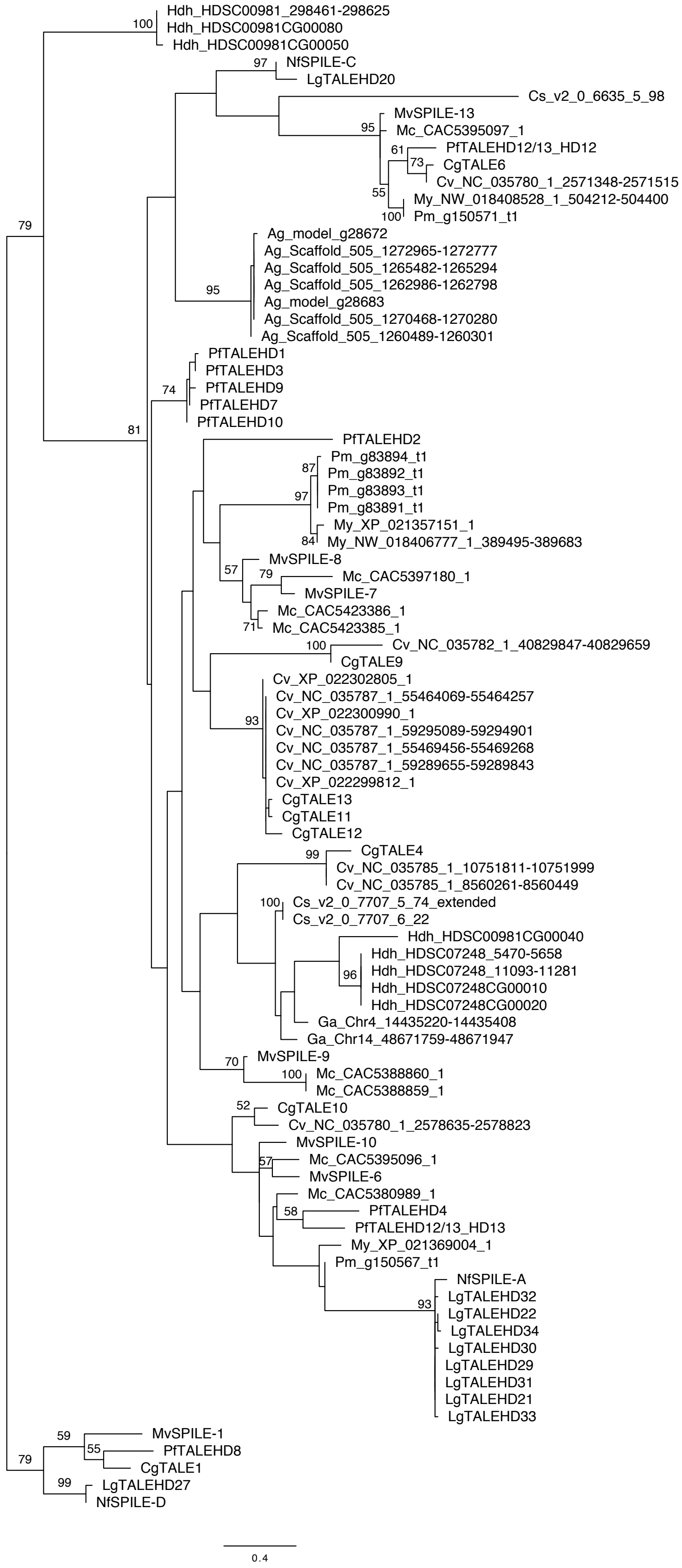

Hdh_HDSC00981_298461-298625
Hdh_HDSC00981CG00080
Hdh_HDSC00981CG00050
NfSPILE-C
LgTALEHD20
Cs_v2_0_6635_5_98
MvSPILE-13
Mc_CAC5395097_1
PfTALEHD12/13_HD12
CgTALE6
Cv_NC_035780_1_2571348-2571515
My_NW_018408528_1_504212-504400
Pm_g150571_t1
Ag_model_g28672
Ag_Scaffold_505_1272965-1272777
Ag_Scaffold_505_1265482-1265294
Ag_Scaffold_505_1262986-1262798
Ag_model_g28683
Ag_Scaffold_505_1270468-1270280
Ag_Scaffold_505_1260489-1260301
PfTALEHD1
PfTALEHD3
PfTALEHD9
PfTALEHD7
PfTALEHD10
PfTALEHD2
Pm_g83894_t1
Pm_g83892_t1
Pm_g83893_t1
Pm_g83891_t1
My_XP_021357151_1
My_NW_018406777_1_389495-389683
MvSPILE-8
Mc_CAC5397180_1
MvSPILE-7
Mc_CAC5423386_1
Mc_CAC5423385_1
Cv_NC_035782_1_40829847-40829659
CgTALE9
Cv_XP_022302805_1
Cv_NC_035787_1_55464069-55464257
Cv_XP_022300990_1
Cv_NC_035787_1_59295089-59294901
Cv_NC_035787_1_55469456-55469268
Cv_NC_035787_1_59289655-59289843
Cv_XP_022299812_1
CgTALE13
CgTALE11
CgTALE12
CgTALE4
Cv_NC_035785_1_10751811-10751999
Cv_NC_035785_1_8560261-8560449
Cs_v2_0_7707_5_74_extended
Cs_v2_0_7707_6_22
Hdh_HDSC00981CG00040
Hdh_HDSC07248_5470-5658
Hdh_HDSC07248_11093-11281
Hdh_HDSC07248CG00010
Hdh_HDSC07248CG00020
Ga_Chr4_14435220-14435408
Ga_Chr14_48671759-48671947
MvSPILE-9
Mc_CAC5388860_1
Mc_CAC5388859_1
CgTALE10
Cv_NC_035780_1_2578635-2578823
MvSPILE-10
Mc_CAC5395096_1
MvSPILE-6
Mc_CAC5380989_1
PfTALEHD4
PfTALEHD12/13_HD13
My_XP_021369004_1
Pm_g150567_t1
NfSPILE-A
LgTALEHD32
LgTALEHD22
LgTALEHD34
LgTALEHD30
LgTALEHD29
LgTALEHD31
LgTALEHD21
LgTALEHD33
MvSPILE-1
PfTALEHD8
CgTALE1
LgTALEHD27
NfSPILE-D
0.4

Fig. S11 Molecular phylogenetic tree of SPILE genes from molluscan species excluding four rapidly evolving SPILE genes and B- and E-clade SPILE genes.

Tree was constructed based on the amino acid sequences of the homeodomains using the maximum likelihood (ML) method. Four rapidly evolving mussel SPILE genes (Mv*SPILE-11*, *-12*, Mc_*CAC5369191*, and Mc_*CAC5369192*) and B- and E-clade SPILE genes were excluded from the dataset for the analysis. The amino acid substitution model was LG + G. The numbers at the nodes are the bootstrap values (only those ≥ 50% and selected are shown). Cg: *Crassostrea gigas*, Pf: *Pinctada fucata*, Lg: *Lottia gigantea*, Nf: *Nipponacmea fuscoviridis,* Mv*: Mytilisepta virgata*. My: *Mizuhopecten yessoensis*, Pm: *Pecten maximus*, Cv: *Crassostrea virginica*, Mc: *Mytilus coruscus,* Hdh: *Haliotis discus hannai*, Ga: *Gigantopelta aegis*, Cs: *Chrysomallon squamiferum*, Ag: *Acanthopleura granulate*.

Fig. S12

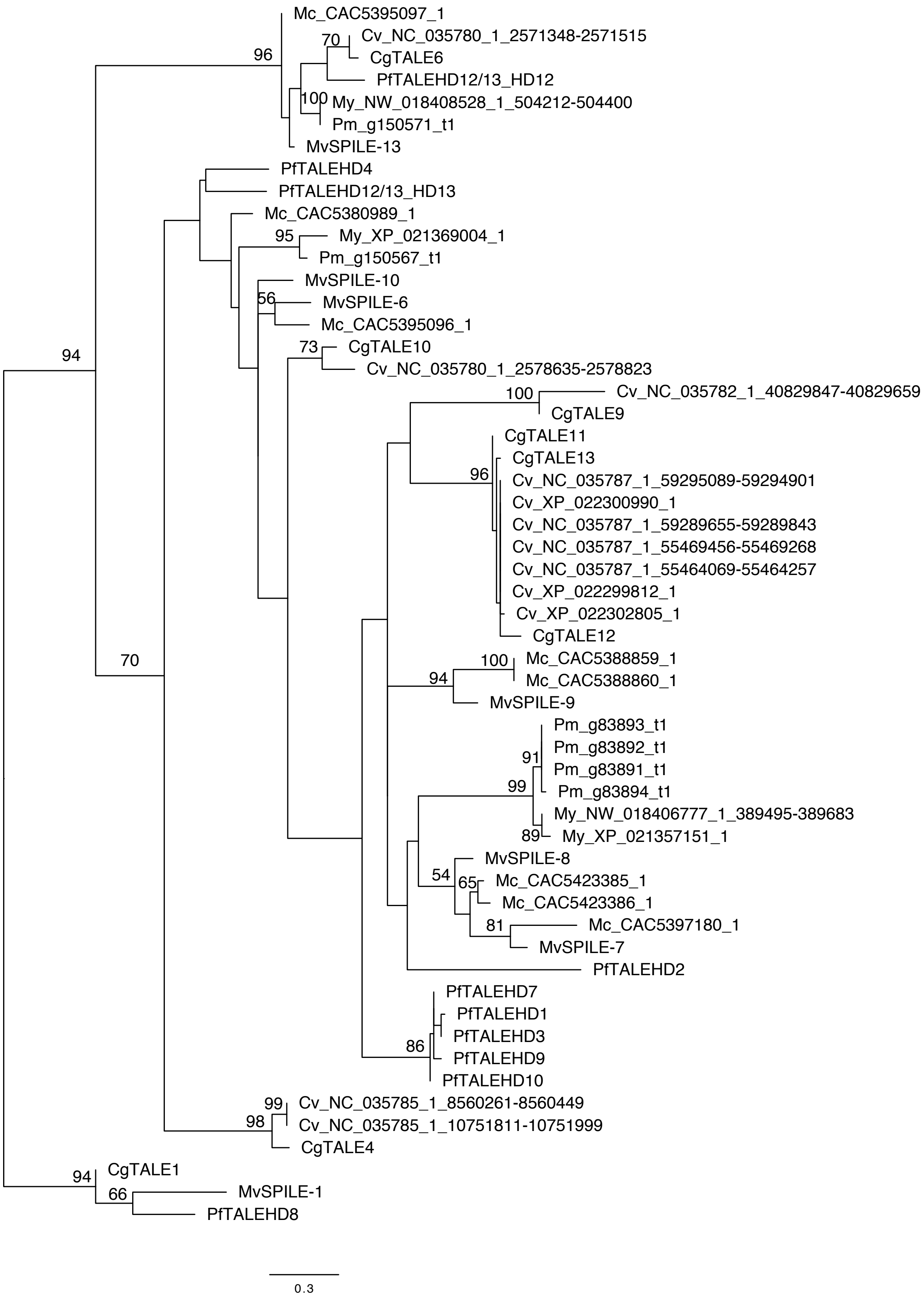
Mc_CAC5395097_1
70
Cv_NC_035780_1_2571348-2571515
96
CgTALE6
PfTALEHD12/13_HD12
100
My_NW_018408528_1_504212-504400
Pm_g150571_t1
MvSPILE-13
PfTALEHD4
PfTALEHD12/13_HD13
Mc_CAC5380989_1
95
My_XP_021369004_1
Pm_g150567_t1
MvSPILE-10
56
MvSPILE-6
Mc_CAC5395096_1
94
73
CgTALE10
Cv_NC_035780_1_2578635-2578823
100
Cv_NC_035782_1_40829847-40829659
CgTALE9
CgTALE11
CgTALE13
96
Cv_NC_035787_1_59295089-59294901
Cv_XP_022300990_1
Cv_NC_035787_1_59289655-59289843
Cv_NC_035787_1_55469456-55469268
Cv_NC_035787_1_55464069-55464257
Cv_XP_022299812_1
Cv_XP_022302805_1
CgTALE12
70
100
Mc_CAC5388859_1
94
Mc_CAC5388860_1
MvSPILE-9
Pm_g83893_t1
Pm_g83892_t1
91
Pm_g83891_t1
99
Pm_g83894_t1
My_NW_018406777_1_389495-389683
89
My_XP_021357151_1
MvSPILE-8
54
65
Mc_CAC5423385_1
Mc_CAC5423386_1
81
Mc_CAC5397180_1
MvSPILE-7
PfTALEHD2
PfTALEHD7
PfTALEHD1
PfTALEHD3
86
PfTALEHD9
PfTALEHD10
99
Cv_NC_035785_1_8560261-8560449
98
Cv_NC_035785_1_10751811-10751999
CgTALE4
94
CgTALE1
66
MvSPILE-1
PfTALEHD8
0.3

Fig. S12 Molecular phylogenetic tree of SPILE genes from bivalve species excluding four rapidly evolving SPILE genes and B- and E-clade SPILE genes.

Tree was constructed based on the amino acid sequences of the homeodomains of bivalve using the maximum likelihood (ML) method. Four rapidly evolving mussel SPILE genes (Mv*SPILE-11*, *-12*, Mc_*CAC5369191*, and Mc_*CAC5369192*) and B- and E-clade SPILE genes were excluded from the dataset for the analysis. The amino acid substitution model was LG + G. The numbers at the nodes are the bootstrap values (only those ≥ 50% and selected are shown). Cg: *Crassostrea gigas*, Pf: *Pinctada fucata*, Mv*: Mytilisepta virgata*. My: *Mizuhopecten yessoensis*, Pm: *Pecten maximus*, Cv: *Crassostrea virginica*, Mc: *Mytilus coruscus,*

Fig. S13

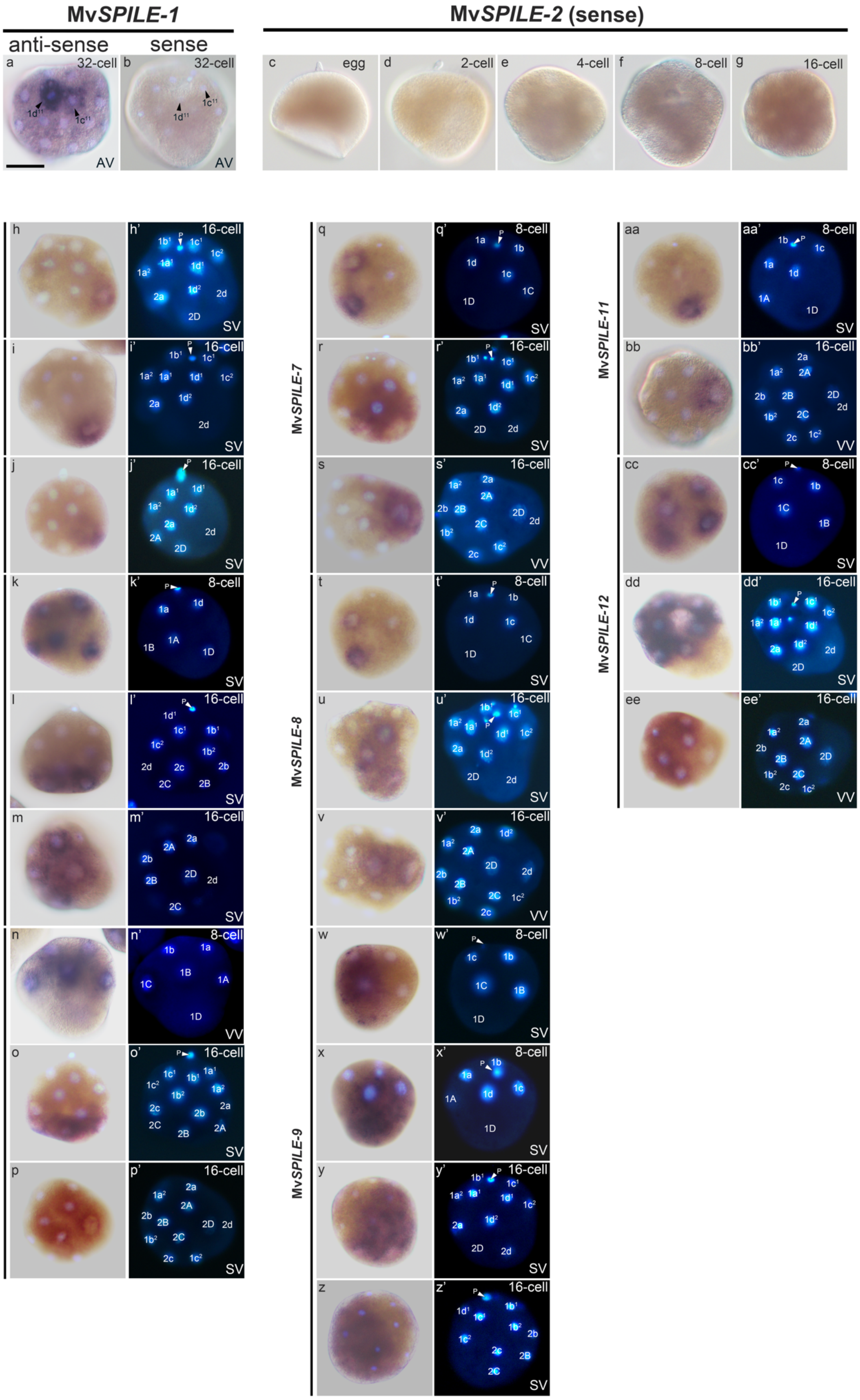

MvSPILE-1
anti-sense
sense
MvSPILE-2 (sense)
a 32-cell
b 32-cell
c egg
d 2-cell
e 4-cell
f 8-cell
g 16-cell
AV
MvSPILE-3
MvSPILE-4
MvSPILE-5
MvSPILE-6
MvSPILE-10
MvSPILE-7
MvSPILE-8
MvSPILE-9
MvSPILE-11
MvSPILE-12
8-cell
16-cell
SV
VV

Fig. S13 Expression patterns of SPILE genes of *Mytilisepta virgata*

(a, b) Expression patterns of Mv*SPILE-1.* (a) Expression of Mv*SPILE-1* was detected in all blastomere, but predominant in $1c^{11}$ and $1d^{11}$ blastomeres at 32-cell stage. (b) Sense probe control for Mv*SPILE-1* at 32-cell stage. No specific expression was detected. (c-g) Sense probe controls for Mv*SPILE-2* from egg to 16-cell stage. No specific expression was detected. (h-ee) Merged images for light field and DAPI shown in Fig. 5-7, without blastomere annotations. (h'-ee') DAPI only images of h-ee. The DNA was stained using DAPI (light blue). Polar bodies are indicated by arrowheads with the letter 'P'.

SV: side view, AV: animal view, VV: vegetal view. Scale bar indicates 50 µm

Reference

Barton-Owen TB, Szabó R, Somorjai IML, Ferrier DEK. A Revised Spiralian Homeobox Gene Classification Incorporating New Polychaete Transcriptomes Reveals a Diverse TALE Class and a Divergent Hox Gene. Genome Biol Evol. 2018;10:2151–67.
